# Tiredness/fatigue and sexuality in everyday life: Findings from an ecological momentary assessment

**DOI:** 10.1007/s00702-025-03008-9

**Published:** 2025-09-11

**Authors:** Hanna M. Mües, Anja C. Feneberg, Charlotte Markert, Urs M. Nater

**Affiliations:** 1 Department of Clinical and Health Psychology, University of Vienna, Liebiggasse 5, Vienna, 1010 Austria; 2 University Research Platform “The Stress of Life – Processes and Mechanisms Underlying Everyday Life Stress”, University of Vienna, Liebiggasse 5, 1010 Vienna, Austria; 3 Department of Social and Preventive Medicine, Center for Public Health, Medical University of Vienna, Vienna, Austria; 4 Clinical Psychology and Psychotherapy for Children and Adolescents, Institute for Psychology, University of Münster, Münster, Germany; 5 Department of Psychotherapy and Systems Neuroscience, Justus-Liebig-University Giessen, Giessen, Germany

**Keywords:** Ecological momentary assessment (EMA), Fatigue, Sexual activity, Sexual arousal, Sexual desire, Tiredness

## Abstract

Tiredness may be associated with increased or decreased sexual experience and behavior while fatigue seems to have a predominantly negative effect, although evidence is scarce. This ecological momentary assessment study is the first to examine associations between tiredness or fatigue and concurrent / subsequent sexual desire or sexual arousal and previous / subsequent sexual activity in daily life, including event-based measurements and considering gender differences. Healthy heterosexual individuals (*n* = 63), aged between 19 and 32 years and in a relationship, indicated their tiredness, general fatigue, physical fatigue, sexual desire, and sexual arousal on an iPod seven times daily over 14 days, and any event-based occurrences of sexual activity. The data were analyzed using multilevel models. Sexual desire was associated with lower concurrent physical fatigue, while sexual desire and arousal were associated with lower (in men) or higher (in women) subsequent physical fatigue. Tiredness was associated with higher subsequent sexual desire and arousal in men only. Although previous physical fatigue was not significantly associated with subsequent sexual activity, cross-level interactions showed that physical fatigue was associated with a lower probability of subsequent sexual activity in men, with a slightly opposing trend in women. The functions and mechanisms underlying tiredness or fatigue and sexual experiences and behavior seem to differ between men and women; this finding suggests that potential interventions should be gender-sensitive in nature. The detected associations between tiredness or fatigue and sexual experiences or behavior highlight the relevance of these factors, and interventions should specifically address tiredness and fatigue as an end-point.

**Supplementary Information:**

The online version contains supplementary material available at 10.1007/s00702-025-03008-9.

## Introduction

Sexuality has a relevant impact on health and well-being (e.g., Anderson 2013), and comprises, among other aspects, sexual experience, including sexual desire and arousal, and sexual behavior (Bancroft 2009; Kaplan 1979; Masters and Johnson 1966). Sexual desire and arousal are closely related, with desire involving motivation and arousal involving excitement (Bancroft 2009). On a physiological level, sexual processes are accompanied by the activation of both the sympathetic and parasympathetic divisions of the autonomic nervous system (Pfaus 2009). In line with this, the Dual Control Model (Bancroft and Janssen 2000; Janssen and Bancroft 2007) proposes sexual processes to depend on balancing elicitation and inhibition of sexual response processes. While gender differences regarding sexual experience and behavior have been discussed, with men tending to report higher levels of sexual desire (Baumeister et al. 2001), actual gender differences may be small or non-existent, with few exceptions (Alexander and Fisher 2003; Dawson and Chivers 2014; Laan et al. 2021; Petersen and Hyde 2010, 2011). Sexual experiences and behavior show diurnal variations, with peaks of sexual desire in the morning and evening (Jankowski et al. 2014; Jocz et al. 2018; Ozdemiroglu 2022). Enhancing positive aspects of sexuality may help improve sexual and overall health and well-being (Anderson 2013; Diamond and Huebner 2012; Satcher 2001).

The demands of daily life utilize physical and psychological resources throughout the day, leading to tiredness or fatigue. Tiredness, a “state of wishing for sleep or rest; weariness” (Oxford University Press, n.d.a), and its more extreme form fatigue, a state of comprehensive exhaustion that results “from mental or physical exertion or illness” (Oxford University Press, n.d.b), are normal reactions that force individuals to rest and thereby replenish their resources (Dörr and Nater 2017). When individuals feel tired or fatigued, such as in the evening after a stressful day, their resources may be too low to fully attend to their romantic partner, especially regarding sexual experiences and behavior.

Tiredness may impair the ability to engage in activities that require effort and thus additional energy (Engle-Friedman 2014). In the context of sexuality, Millar et al. (2019) called this the “too tired to have sex” phenomenon. Alternatively, tiredness may impair self-regulation abilities in the face of a choice or temptation, such as an opportunity for sexual activity, which Millar et al. (2019) called the “not too tired to have sex, but too tired to be proactive and vigilant about sexual health” phenomenon. In terms of physiological processes, associations between tiredness and the autonomic nervous system remain unclear (Bisogni et al. 2017; Chen et al. 2022; Donadio et al. 2007; Kasai et al. 2020; Lombardi et al. 2008; Pressman and Fry 1989; Taranto Montemurro et al. 2014), although one study suggested an association of increased sympathetic nervous system activity with objective but not subjective excessive daytime sleepiness in men with obstructive sleep apnea (Chen et al. 2022). However, fatigue has been associated with an increase in sympathetic nerve activity and a decrease in parasympathetic nerve activity in the autonomic nervous system (Tanaka et al. 2015). Furthermore, previous studies hint at gender differences in reported tiredness and fatigue. In particular, women tend to report tiredness as well as fatigue more frequently than men (Bensing et al. 1999; Kenter et al. 2003; Meeuwesen et al. 2002). Furthermore, similar to sexual experiences and behavior, reported levels of tiredness vary throughout the day, with higher levels in the morning and evening (Crowe et al. 2019; Dockray et al. 2010; Stone et al. 1996, 2006), and reported fatigue levels show a similar diurnal pattern (Powell et al. 2017; Taub and Berger 1974).

A better understanding of the link between tiredness or fatigue and sexual experience and behavior in daily life may allow for the enhancement of positive aspects of sexuality and foster the right for “pleasurable and safe sexual experiences” (World Health Organization 2006, p.5).

A study conducted in France found that tiredness was the most frequently mentioned reason for not having more sex, in both women (43%) and men (36%) (Colson et al. 2006). Similarly, in a U.S. study, 33% of men reported a decrease in sexual desire due to tiredness, although in the same study, 29% of men reported an increase in sexual desire due to tiredness, with this latter group being younger on average (Millar et al. 2019).

Fatigue, which should be differentiated as a more extreme form of tiredness (Finsterer and Mahjoub 2014), has been associated with negative effects on sexuality and the occurrence of sexual problems and dysfunctions (e.g. Gilhooly et al. 2001). For instance, in a study with married women in Singapore using biweekly diaries, Tan (2021) found a negative effect of fatigue on the frequency of sexual activities.

Hence, previous studies have yielded inconclusive findings, although the majority point to a negative association between tiredness or fatigue and sexual experience and behavior. Nevertheless, the direction of these effects remains to be investigated. The evidence in this area is scarce, and the studies conducted so far encompass a number of limitations and did not investigate bidirectional associations between tiredness or fatigue and sexual experience and behavior. In particular, the research to date has focused especially on patients suffering from chronic fatigue syndrome. While it is important to investigate the associations between CFS and sexuality, it is essential to study the association between fatigue and sexuality in healthy individuals as well, as such research may provide fundamental knowledge on basic mechanisms underlying the association between tiredness or fatigue and sexuality in daily life as well as further insights to improve sexuality in daily life. Moreover, studies investigating associations between tiredness or fatigue and sexuality have largely focused on women, with research examining this link in men and considering gender differences remaining scarce. It is important to consider gender differences in this context, and to differentiate between tiredness and fatigue (Finsterer and Mahjoub 2014), with the latter including both mental and physical aspects. While general fatigue has been considered in previous studies, physical fatigue has not been explicitly investigated in this context. In order to examine the association between both mental and physical aspects of fatigue and sexuality, an assessment of general and physical fatigue is necessary. Furthermore, previous studies show methodological limitations as they mostly investigated associations between tiredness or fatigue and sexuality using cross-sectional designs with retrospective questionnaires, which are prone to recall bias and do not allow for insights into the dynamic relationships between tiredness or fatigue and sexuality as they occur in real life (Shiffman et al. 2008). One exception is the study by Tan (2021), which was conducted longitudinally using biweekly diaries and focused on effects of fatigue on sexual experience and behavior but did not investigate the reverse association, namely effects of sexual experience and behavior on tiredness or fatigue. The authors were unable to investigate the sequence of events in time more precisely or identify changes over the course of one day, as the data collection was limited to twice a week (Shiffman et al. 2008; Tan 2021). As described above, research that considers variations in tiredness, fatigue, and sexual experiences and behavior throughout the day, additionally examining bidirectional associations, is needed.

In summary, bidirectional associations between tiredness, general fatigue or physical fatigue and sexual experiences and behavior in healthy individuals in daily life, considering gender differences, remain unclear. Therefore, longitudinal studies exploring variations of these associations within one day are needed. The present paper aims to address these research gaps by investigating bidirectional associations between tiredness, general fatigue, or physical fatigue and sexual desire, sexual arousal, or sexual activity in healthy women and men. Ecological Momentary Assessment (EMA) encompasses data collection in the participant’s everyday life over a specific period of time and at previously defined moments, and thereby minimizes recall bias while maximizing ecological validity (Shiffman et al. 2008). As such, EMA studies include multiple measurements per day, as well as event-based entries, enabling variations throughout the day to be mapped. The inclusion of event-based entries provides the unique advantage of examining sexual behavior in the form of sexual activities as they occur in daily life, as there may be associations with tiredness, general fatigue or physical fatigue in addition to sexual experience.

The present study is the first to investigate the association between tiredness, general fatigue or physical fatigue and concurrent / subsequent sexual desire or arousal and previous / subsequent sexual activity bidirectionally in healthy women and men in daily life, using multiple measurements per day as well as measurements directly after sexual activities. So far, previous literature mostly suggests negative associations between tiredness/fatigue and sexual desire/sexual arousal/sexual activity although a different methodological approach was applied. Based on previous findings, with regard to concurrent associations, we hypothesized negative associations between 1) tiredness/fatigue and concurrent sexual desire/sexual arousal/sexual activity and vice versa. In particular, we hypothesize negative associations between 1a) tiredness and sexual desire, and vice versa, 1b) tiredness and sexual arousal, and vice versa, 1c) general fatigue and sexual desire, and vice versa, 1d) general fatigue and sexual arousal, and vice versa, 1e) physical fatigue and sexual desire, and vice versa, and 1f) physical fatigue and sexual arousal, and vice versa. With regard to associations over time, we hypothesized negative associations between (2) tiredness/fatigue and subsequent sexual desire/sexual arousal/sexual activity and vice versa. More specifically, the same hypotheses were derived as for hypothesis 1) (hypotheses 2a to 2f). Additionally, we hypothesized a negative association between 3) tiredness/fatigue and sexual desire/sexual arousal/sexual activity and vice versa. In particular, we hypothesized a negative association between tiredness (3a), general fatigue (3b), or physical fatigue (3c) and previous / subsequent sexual activity. Finally, we hypothesized gender differences for hypotheses 1a to 3c, such that associations would be stronger in women compared to men.

## Method

For the present study, we conducted a secondary analysis using data from an EMA study on stress and sexuality (see Mües et al. (in press) for details on recruitment, inclusion and exclusion criteria, procedure, and further study details) that was based on work from Mües et al. (2025). Research data and analysis code are available upon request.

### Participants

Recruitment took place between 2018 and 2020 in Vienna, Austria. Participants were aged between 18 and 35 years (to prevent influences due to age-related changes (Roberts et al. 2021), German-speaking, and healthy (i.e., no chronic somatic illnesses, no over-/underweight, no alcohol or drug consumption, no mental disorder to prevent influences due to ill-health). Furthermore, participants did not take any psychiatric medication or medication influencing hormones or sexuality and did not have children. In women, the phase of the menstrual cycle was controlled for. All participants had been in a heterosexual relationship for at least one year and only one partner of a dyad was included in the study.

### Measures

At the baseline assessment, participants provided data including their self-identified gender (0 = men, 1 = women), age, age of first sexual contact (in years), importance of sexuality, and sex life satisfaction, and completed the Multidimensional Fatigue Inventory (MFI-20; Smets et al. 1995).

Longer questionnaires have been associated with an increased burden and a negative effect on data quantity and quality (Eisele et al. 2020). At each measurement time point during the EMA phase, we therefore kept the questionnaire length to a minimum by using a single-item approach. This approach is advantageous in longitudinal studies for unidimensional constructs as it is less time-consuming and eliminates item redundancy (Robins et al. 2001). Fatigue, for example, can be validly assessed using a single item (van Hooff et al. 2007).

### Tiredness

To measure current tiredness during the EMA phase, we chose a single item from the alertness/tiredness scale from the German version of the Multidimensional Mood Questionnaire (MDBF, short form A, 12 items; Steyer et al. 1997) for reasons of face validity. Six times per day, participants rated their momentary tiredness using the item “At the moment, I feel tired” on a 5-point scale from (= no tiredness) to 5 (= high tiredness); for the purpose of analysis, 1 was recoded to zero and 5 was recoded to 4 (Steyer et al. 1997).

### Fatigue

At the baseline assessment the EMA phase, fatigue was measured using the German version of the Multidimensional Fatigue Inventory (MFI), which consists of the five dimensions General Fatigue, Physical Fatigue, Mental Fatigue, Reduced Motivation, and Reduced Activity (MFI-20, 20 items; Smets et al. 1995). During the EMA phase, general fatigue was measured using a single item based on the MFI-20, which was chosen due to face validity and has previously been used as an indicator of fatigue by Doerr et al. (2015). The formulated item “At the moment, I feel fatigued” was assessed six times per day on a 5-point scale from 1 (= no general fatigue) to 5 (= high general fatigue), which was recoded to a range from 0 to 4 for the analyses (Smets et al. 1995). Physical fatigue was likewise measured using a single item from the MFI-20 (Smets et al. 1995), which was adapted to the EMA design of this study. The item “At this moment, physically, I can take on a lot” (Lin et al. 2009, “Table 4”) was chosen as it seemed most suitable for our design. This item was rated six times per day on a 5-point scale from 1 (= high physical fatigue) to 5 (= low physical fatigue). For the analyses, the variable was recoded such that 0 indicated low physical fatigue and 4 indicated high physical fatigue.

### Sexual desire, arousal, and activity

Information on sexual desire and arousal during the EMA phase was collected using single items rated on 5-point scales (1 indicating absence of sexual desire or arousal since the last time point, recoded to 0 for the analyses; 5 indicating very strong presence of sexual desire or arousal since the last time point, recoded to 4) six times per day (excluding the time point directly after awakening). Event-based measurements occurred directly after sexual activity, i.e. masturbation, petting, oral sex, sexual intercourse, and/or anal sex. Occurrence of an event was time-lagged to the next measurement time point, indicating previous sexual activity, and reverse time-lagged to the previous measurement time point, indicating subsequent sexual activity (0 = no previous/subsequent sexual activity, 1 = previous/subsequent sexual activity). Any additional events that had taken place during the night and not been recorded as events were assessed at the awakening measurement.

### Possible confounders

As variance in tiredness, general fatigue, physical fatigue, sexual desire, sexual arousal, and sexual activity can be influenced by age, depressive symptoms, relationship duration (in months), relationship quality ([relationship questionnaire], PFB, (Hahlweg 1979), time since awakening (in hours), sleep quality, and occurrence of previous sexual activity (unless included as predictor or outcome), these were included as possible confounders and controlled for. Furthermore, autocorrelation (the outcome value of the previous measurement time point was included in the respective model) was controlled for, with the exception of models with sexual activity as an outcome. Depressive symptoms were assessed at the baseline assessment using the scale on depressive symptoms (PHQ-9) from the German version of the Patient Health Questionnaire (PHQ-D) (Löwe et al. 2002; Spitzer et al. 1999). During the EMA phase, time since awakening (in hours) was assessed as the time difference between participants’ reported awakening time on a particular day and the time of the measurement recorded on the iPod. Sleep quality was assessed using a single item from the German version of the Pittsburgh Sleep Quality Index (Backhaus et al. 2002; Buysse et al. 1989), assessing how restful participants would rate their sleep during the last night overall on a scale from 1 (= not at all restful) to 5 (= very restful). Occurrence of sexual activity was assessed using the event-based measurements (0 = no previous sexual activity, 1 = previous sexual activity).

### Procedure

Following a baseline assessment, participants entered data into an iPod provided for the purpose of the study seven times per day (upon awakening, 30 min after awakening, 11am, 2pm, 5pm, 8pm, before going to sleep) and provided event-based entries within 15 min of any sexual activity for 14 days. Following this, participants returned to the lab for a post-monitoring interview. For full study participation, participants received study compensation of €100. The study received ethical approval by the Ethics Committee of the University of Vienna, Austria (reference number: 00308), and all participants and their partners gave informed consent prior to study participation. Consent from partners was required as data on their sex life was collected via the participants whenever the participants reported sexual activity with their partner.

### Statistical analysis

For descriptive statistics, we report mean, standard deviation, count, and range. For baseline data preparation, we used IBM SPSS Statistics 26. For data preparation, creating tables and graphs, and conducting calculations, we used Excel 2016, IBM SPSS Statistics 26, R 4.2.2 (R Core Team 2022), and R studio (RStudio Team 2022) and the R packages haven (Wickham et al. 2022), lubridate (Grolemund and Wickham 2011), psych (Revelle 2022), gtsummary (Sjoberg et al. 2021), tidyverse (Wickham et al. 2019), flextable (Gohel and Skintzos 2022), Rmisc (Hope 2022), ggplot2 (Wickham 2016), lme4 (Bates et al. 2015), jtools (Long 2022), performance (Lüdecke et al. 2021), lmerTest (Kuznetsova et al. 2017), and sjPlot (Lüdecke 2022). Measurement time points were included if they occurred within ± 1 h of the intended time point. Due to the nested structure of the data, we calculated binomial generalized linear mixed-effects models (for subsequent occurrence of sexual activity as a dichotomous outcome) or linear mixed-effects models (for all other outcomes) with random intercepts and slopes. Direct effects and cross-level interactions with gender were investigated. Two- or three-level models were calculated depending on intraclass correlation coefficients (ICC; see Results); two- and three-level models were compared using ANOVAs as well as sufficient random effect variance. While dichotomous variables were uncentered on all levels, continuous variables on the observation level (level 1) were group mean centered, those on the participant level (level 2 or 3) were grand mean centered, and time since awakening was centered around the time the participant awoke (Enders and Tofighi 2007; Nezlek and Mroziński 2020). Concurrent data as well as time-lagged data (the predictor was lagged from one measurement time point to the next time point of the same day) associations were examined whenever possible. Associations between tiredness, general fatigue or physical fatigue and sexual desire, sexual arousal, or sexual activity were examined bidirectionally, and in each model, the association between one predictor and one outcome was analyzed, considering confounding variables. Time points with missing values in the model-relevant variables were excluded for each model separately. For analysis, a null model was calculated (outcome, random intercept), followed by a random intercept and fixed slope model (predictor, confounding variables), a random intercept and random slope model (random slope in the form of the predictor was added unless there was no sufficient variance) (Baird and Maxwell 2016), a cross-level interaction model (cross level-interaction with gender was added), and the final model (Aguinis et al. 2013; Schwartz 2022). Inclusion of fixed effects and of the interaction was assessed using full maximum likelihood, while restricted maximum likelihood was used for all other models (Schwartz 2022). For significance tests, Satterthwaite’s method was used (Kuznetsova et al. 2017) and the significance level was set at *p* < 0.05. Goodness of model fit is reported using marginal R^2^ and conditional R^2^ (Nakagawa and Schielzeth 2013). For cross-level-effect calculations, a sample size of 50 or more participants has been deemed sufficient (Maas and Hox 2005).

## Results

Sixty-three healthy heterosexual individuals were included in the study (32 women, 31 men, *M*_age_ = 24.51, *SD*_age_ = 2.99, range_age_: 19–32 years). Of 6,174 possible measurement time points during the EMA phase, 12.6% were missing (sample characteristics and missing data are described in more detail in Mües et al. (in press). At the baseline assessment, mean general and physical fatigue scores were lower compared to norms of the MFI-20 subscores for each gender and for the total sample (Westenberger et al. 2022) (see Table S1 in the Online Resource 1).

**Fig. 1:**
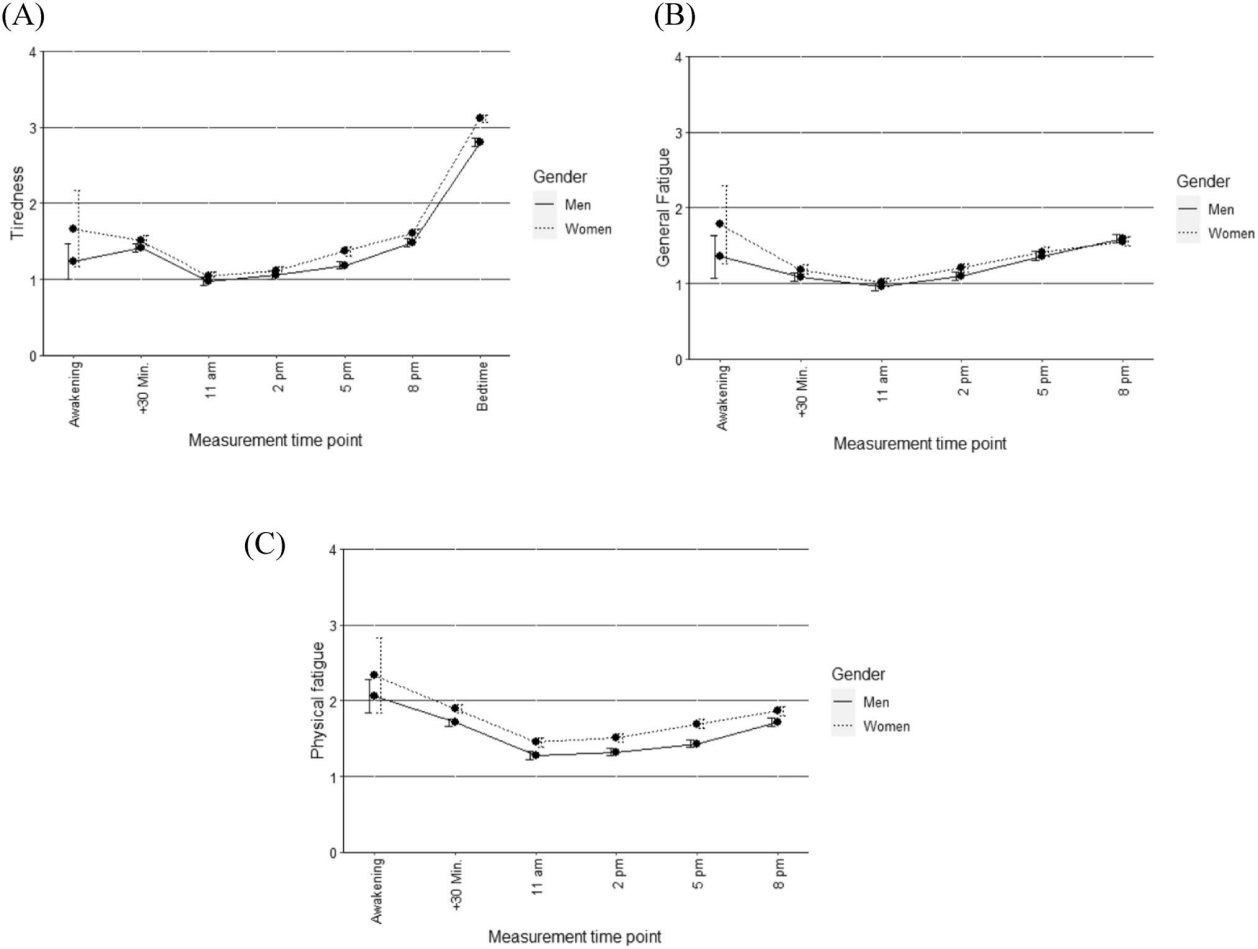
Diurnal patterns of **A** tiredness, **B** general fatigue, and **C** physical fatigue over the course of the day. Higher values indicate higher levels of the construct. Error bars indicate the standard error of the mean

Levels of tiredness, general fatigue, physical fatigue, sexual desire, and sexual arousal during the EMA phase are described in the main text or presented in Table S2 in the Online Resource 1. The diurnal patterns of tiredness, general fatigue, and physical fatigue are shown in Fig. 1. Participants reported slightly elevated tiredness in the morning, which decreased at around 11am and then increased again until bedtime. General and physical fatigue showed a similar pattern over the day, though neither increased as much as tiredness in the evening. Women reported slightly higher tiredness, general fatigue, and physical fatigue compared to men, with physical fatigue showing the largest difference. Diurnal patterns of sexual desire and arousal varied at awakening and increased slightly in the evening (a graphical depiction is available in Mües et al. (in press)). Women tended to report lower levels of sexual desire and arousal than did men. On average, participants reported 421 (*M* = 6.68, *SD* = 4.53, range = 1–32; men: *M* = 8.16, *SD* = 5.63, range: 2–32; women: *M* = 5.25, *SD* = 2.46, range = 1–10) sexual activities as event-based measurements during the EMA phase (further details are provided in Mües et al. (in press)).

Table 1 shows intraclass correlation coefficients for models with the outcomes tiredness, general fatigue, physical fatigue, sexual desire, and sexual arousal. Three-level models were calculated for models with sexual desire and arousal as an outcome and two-level models were calculated for models with tiredness, general fatigue, physical fatigue, and sexual activity as an outcome. Considering model assumptions, multicollinearity was assessed using the Variance Inflation Factor (VIF), and all predictors showed VIF values below 5, indicating no problematic multicollinearity. Furthermore, potential outliers were examined, and no influential outliers were detected. Results of multilevel models are shown in Tables S3 to S32 (see Online Resource 1).

**Table 1:** Intraclass correlation coefficients for tiredness, general fatigue, physical fatigue, sexual desire, and sexual arousal as outcomes

Outcome	ICC (2-level)	ICC (3-level)	
	Participant-level	Day-level	Participant-level
Tiredness^a^	**0.15**	0.05	0.15
General fatigue^a^	**0.21**	0.08	0.20
Physical fatigue^a^	**0.20**	0.08	0.20
Sexual desire^b^	0.14	**0.11**	**0.13**
Sexual arousal^b^	0.13	**0.08**	**0.13**
Subsequent sexual activity^a^	**0.05**	< 0.01	0.05

*ICC* intraclass correlation coefficient

^a^Two-level models were calculated; the corresponding ICC is marked in bold

^b^Three-level models were calculated; the corresponding ICC is marked in bold

### Hypothesis 1:

Negative bidirectional associations between tiredness/fatigue and sexual desire/sexual arousal/sexual activity at the same measurement point.

### Hypothesis 1a:

There was no significant association between tiredness and concurrent sexual desire (*p* = 0.109) or vice versa (*p* = 0.117), and there were no significant cross-level interactions with gender (*p* = 0.879, *p* = 0.280, respectively).

### Hypothesis 1b:

There was no significant association between tiredness and concurrent sexual arousal (*p* = 0.256) or vice versa (*p* = 0.152), irrespective of gender (*p* = 0.882, *p* = 0.430, respectively).

### Hypothesis 1c:

There was no significant association between general fatigue and concurrent sexual desire (*p* = 0.414) or vice versa (*p* = 0.415), irrespective of gender (*p* = 0.688, *p* = 0.188, respectively).

### Hypothesis 1d:

There was no significant association between general fatigue and concurrent sexual arousal (*p* = 0.129) or vice versa (*p* = 0.078), irrespective of gender (*p* = 0.235, *p* = 0.797, respectively).

### Hypothesis 1e:

Physical fatigue was not significantly associated with concurrent sexual desire (*p* = 0.079) and there was no significant cross-level interaction with gender (*p* = 0.580). Higher sexual desire was significantly associated with lower concurrent physical fatigue (*UC* = -0.04, *p* = 0.048, *Marginal R²* = 0.243, *Conditional R²* = 0.426), while there was no significant cross-level interaction with gender (*p* = 0.487).

### Hypothesis 1f:

There was no significant association between physical fatigue and concurrent sexual arousal (*p* = 0.328) or vice versa (*p* = 0.159), irrespective of gender (*p* = 0.190, *p* = 0.624, respectively).

### Hypothesis 2:

Negative bidirectional associations between tiredness/fatigue and subsequent sexual desire/sexual arousal/sexual activity from one measurement time point to the next.

### Hypothesis 2a:

**Fig. 2:**
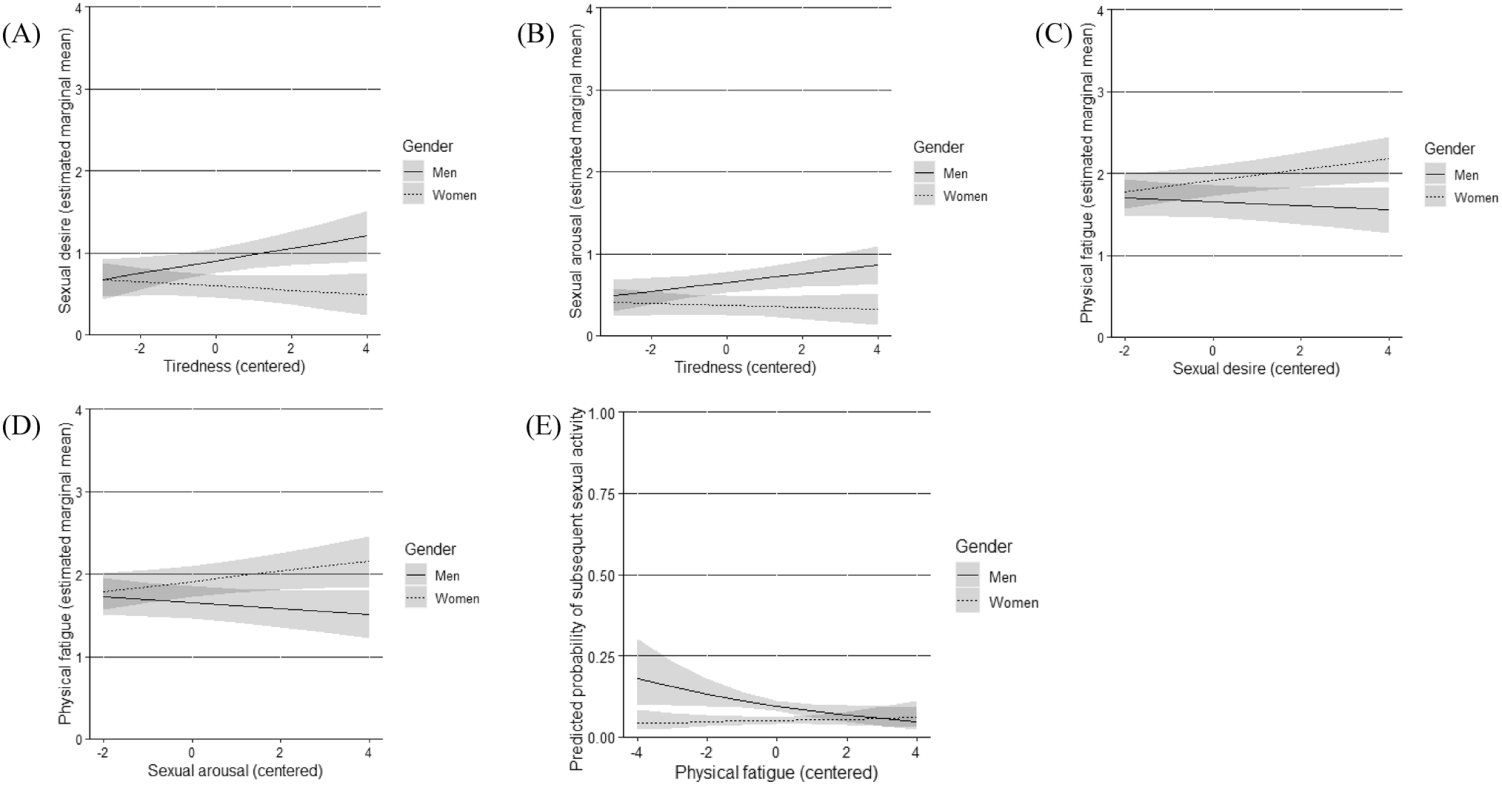
Cross-level interactions with gender for **A** three-level model on the effect of tiredness on subsequent sexual desire, **B**, three-level model on the effect of tiredness on subsequent sexual arousal, **C** two-level model on the effect of sexual desire on subsequent physical fatigue, **D** two-level model on the effect of sexual arousal on subsequent physical fatigue, and **E** two-level model on the effect of physical fatigue on occurrence of subsequent sexual activity. Higher values indicate higher levels of the construct

Higher tiredness was significantly associated with higher subsequent sexual desire (*UC* = 0.08, *p* = 0.023, *Marginal R²* = 0.120, *Conditional R²* = 0.295); however, there was a significant cross-level interaction with gender (*UC* = -0.10, *p* = 0.017). Figure 2A shows that the association is positive for men, i.e., higher tiredness was associated with higher subsequent sexual desire, however, for women, it is slightly negative, i.e., higher tiredness was associated with lower subsequent sexual desire. Sexual desire was not significantly associated with subsequent tiredness (*p* = 0.174) and there was no significant cross-level interaction with gender (*p* = 0.077).

### Hypothesis 2b:

Higher tiredness was significantly associated with higher subsequent sexual arousal (*UC* = 0.05, *p* = 0.032, *Marginal R²* = 0.160, *Conditional R²* = 0.307), but again, a significant cross-level interaction with gender emerged (*UC* = -0.06, *p* = 0.036). As depicted in Fig. 2B, this association was positive in men, i.e., higher tiredness was associated with higher subsequent sexual arousal, but slightly negative in women, i.e., higher tiredness was associated with lower subsequent sexual arousal. Sexual arousal was not significantly associated with subsequent tiredness (*p* = 0.835) and there was no significant cross-level interaction with gender (*p* = 0.069).

### Hypothesis 2c:

There was no significant association between general fatigue and subsequent sexual desire (*p* = 0.882) or vice versa (*p* = 0.997), irrespective of gender (*p* = 0.579, *p* = 0.582, respectively).

### Hypothesis 2d:

There was no significant association between general fatigue and subsequent sexual arousal (*p* = 0.655) or vice versa (*p* = 0.587), irrespective of gender (*p* = 0.977, *p* = 0.979, respectively).

### Hypothesis 2e:

Physical fatigue was not significantly associated with subsequent sexual desire (*p* = 0.179) and there was no significant cross-level interaction with gender (*p* = 0.476). Sexual desire was not significantly associated with subsequent physical fatigue (*p* = 0.302); however, there was a significant cross-level interaction with gender (*UC* = 0.09, *p* = 0.008). As shown in Fig. 2C, this association was positive in women, i.e., higher sexual desire was associated with higher subsequent physical fatigue, but negative in men, i.e., higher sexual desire was associated with lower subsequent physical fatigue.

### Hypothesis 2f:

Physical fatigue was not significantly associated with subsequent sexual arousal (*p* = 0.082) and there was no significant cross-level interaction with gender (*p* = 0.186). Sexual arousal was not significantly associated with subsequent physical fatigue (*p* = 0.162), but there was a significant cross-level interaction with gender (*UC* = 0.10, *p* = 0.015). As shown in Fig. 2D, this association was positive in women, i.e., higher sexual arousal was associated with higher subsequent physical fatigue, but negative in men, i.e., higher sexual arousal was associated with lower subsequent physical fatigue.

### Hypothesis 3:

Negative bidirectional associations between tiredness/fatigue and sexual desire/sexual arousal/sexual activity and vice versa.

### Hypothesis 3a:

There was no significant association between previous sexual activity and subsequent tiredness (*p* = 0.621) or between previous tiredness and subsequent sexual activity (*p* = 0.085), irrespective of gender (*p* = 0.866, *p* = 0.768).

### Hypothesis 3b:

There was no significant association between previous sexual activity and subsequent general fatigue (*p* = 0.053) or between previous general fatigue and subsequent sexual activity (*p* = 0.643), irrespective of gender (*p* = 0.718, *p* = 0.946).

### Hypothesis 3c:

There was no significant association between previous sexual activity and subsequent physical fatigue (*p* = 0.695), irrespective of gender (*p* = 0.586). There was no significant association between previous physical fatigue and subsequent sexual activity (*OR* = 0.93, *p* = 0.261). However, when including the cross-level interaction with gender, previous physical fatigue was significantly associated with a lower probability of occurrence of subsequent sexual activity (*OR* = 0.83, *p* = 0.030, *Marginal R²* = 0.037, *Conditional R²* = 0.052), with a significant cross-level interaction with gender (*OR* = 1.26, *p* = 0.048) showing that higher physical fatigue was associated with a lower probability of subsequent sexual activity in men, while women showed a slightly opposing trend (Fig. 2E).

## Discussion

This study aimed to investigate associations between tiredness, general fatigue, or physical fatigue and sexual desire, sexual arousal, or sexual activity in healthy men and women in everyday life. Overall, the findings indicate some bidirectional associations between tiredness/fatigue and sexual desire/sexual arousal/sexual activity, as well as the relevance of considering gender in the associations. More specifically, the results show little support for hypothesis 1, which proposed negative bidirectional associations between tiredness/fatigue and concurrent sexual desire/sexual arousal/sexual activity. In particular, while higher sexual desire was associated with lower concurrent physical fatigue, the other associations were insignificant. Considering hypothesis 2, which suggested negative bidirectional associations between tiredness/fatigue and subsequent sexual desire/sexual arousal/sexual activity, the findings provide some support. More specifically, higher tiredness was associated with higher subsequent sexual desire/sexual arousal. However, when considering gender, this association remained positive for men but showed a slightly negative trend in women. Furthermore, while sexual desire/sexual arousal was not significantly associated with subsequent physical fatigue, the association shows a positive trend in women and a negative trend in men, thereby potentially explaining the insignificant result of the overall association. Moreover, the findings provide some support for hypothesis 3, i.e., negative bidirectional associations between tiredness/fatigue and sexual desire/sexual arousal/sexual activity. In particular, previous physical fatigue was significantly associated with a lower probability of occurrence of subsequent sexual activity and the association showed a negative trend in men, but a slightly opposing trend in women. The results are discussed in further detail below.

The results did not reveal any concurrent associations between tiredness and sexual desire and arousal or vice versa, in contrast to hypotheses 1a and 1b. However, over time, i.e., from one measurement time point to the next within the same day, higher tiredness was associated with higher subsequent sexual desire and arousal, although cross-level interactions with gender showed that while this was the case for men (in contrast to hypotheses 2a and 2b), it did not apply to women. Instead, women showed a trend for a negative association, insofar as higher tiredness was associated with lower subsequent sexual desire and arousal (in line with hypotheses 2a and 2b). These results underline the relevance of examining these associations over-time as associations appear to be primarily time-lagged. When discussing these results in the light of the literature, it should be kept in mind that previous studies used a different methodology to the present study, as no previous research employed an EMA design with such close monitoring of changes over the course of the day. The positive association found in men in the present study is partly in line with Millar et al. (2019), who reported a positive association in some (younger) men and a negative association in other men in their sample. The negative association in women in the present study corresponds to the findings of Colson et al. (2006), although the latter authors also found this trend for men, in contrast to our findings. On a physiological level, the positive association in men may hint at a link between tiredness and sympathetic nervous system activation. This notion would be supported by Lorenz et al. (2012), who revealed associations between moderate activation of the sympathetic nervous system and higher genital arousal in women. Nevertheless, further research is required in order to draw definitive conclusions in this regard. Moreover, the positive association in men may further be explained by excitation transfer, as proposed by Zillmann (1983), according to which arousal (potentially the case with tiredness) may trigger sexual arousal when faced with a stimulus perceived as sexual (Bancroft et al. 2003). Further research into gender differences in the association of tiredness with sexual desire and arousal seems advisable in order to gain further insights. Sexual desire and arousal did not show an effect on subsequent tiredness.

Furthermore, we detected no significant associations between general fatigue and sexual desire and arousal, or vice versa, either at the same time point or over time (in contrast to hypotheses 1c, 1d, 2c, and 2d), and no associations between physical fatigue and concurrent or subsequent sexual desire or arousal (in contrast to hypotheses 1e, 1f, 2e, and 2f). These findings contradict previous studies which reported negative associations between fatigue and sexuality-related factors (Alia et al. 2019; Bazzichi et al. 2012; Blazquez et al. 2008, 2015; Gilhooly et al. 2001; Tan 2021; Vermeulen and Scholte 2004). However, as mentioned above, previous research applied different methodological approaches compared to the present study, and additionally, most (though not all) of these studies investigated only women and only considered pathological forms of fatigue, which may at least partly explain the contrasting results.

Higher sexual desire, but not higher sexual arousal, was associated with lower concurrent physical fatigue (thus supporting hypothesis 1e but not 1f). Additionally, although there was no significant time-lagged association of sexual desire and arousal with subsequent physical fatigue, cross-level interactions with gender revealed contrasting associations for men and women. Higher sexual desire and arousal were associated with lower subsequent physical fatigue in men (supporting hypotheses 2e and 2f) but with higher subsequent physical fatigue in women (in contrast to hypotheses 2e and 2^2^). Accordingly, it appears that while sexual desire may have energizing properties, especially in men, it may lead to physical fatigue over time in women. However, these links warrant further investigation and should be interpreted with caution, as previous studies did not explicitly investigate the link between physical fatigue and sexuality.

Our findings showed that previous sexual activity was not associated with subsequent tiredness or general fatigue or vice versa (in contrast to hypotheses 3a and 3b) and was likewise not associated with physical fatigue (in contrast to hypothesis 3c). However, previous physical fatigue was associated with a lower probability of subsequent sexual activity in men (in line with hypothesis 3c), while women showed a slightly opposing trend (in contrast to hypothesis 3c). There is no previous research investigating these associations in daily life with such a high frequency of measurements, and studies should therefore investigate these links further, taking gender differences into account.

In terms of gender differences, tiredness was associated with higher subsequent sexual desire and arousal in men but lower subsequent sexual desire and arousal in women. Moreover, sexual desire and arousal were associated with lower subsequent physical fatigue in men but higher subsequent physical fatigue in women. Furthermore, while physical fatigue was associated with a lower probability of subsequent sexual activity in men, a slightly opposing trend emerged in women. Hence, tiredness may be a factor that hinders subsequent sexual processes in women but facilitates them in men, whereas physical fatigue seems to hinder subsequent sexual activity in men but to slightly facilitate it in women. These gender differences hint at different processes in men and women in terms of associations between tiredness or physical fatigue and sexual desire, sexual arousal, or sexual activity, and need to be investigated further.

### Limitations

Due to self-selection bias as well as the narrow inclusion and exclusion criteria of the study, the present sample presents a clearly defined specific part of the population and is not representative of the general population, and the results cannot be generalized. As such, this study does not contain data on gender and sexual minorities. Moreover, the present study is a secondary analysis using data from a study investigating the link between stress and sexuality, meaning that the present research question was not the primary aim of the larger study. Nevertheless, all relevant information was collected to also investigate the hypotheses examined in this paper. Additionally, the use of single items during the EMA phase may have increased measurement error, contributing to low ICCs and underestimating trait-level variability. At the same time, this approach is advantageous as it is less time-consuming and eliminates item redundancy (Robins et al. 2001), thereby reducing participant burden. Moreover, the design of the present study only allows conclusions regarding associations, but it does not allow for any causal conclusions, which should be kept in mind when interpreting the findings. Furthermore, it is unclear if the distinction between tiredness and fatigue was apparent to the participants, who may use a different definition than clinical samples. Similarly, the distinction between sexual desire and arousal, which are distinct (McCabe et al. 2016; Parish et al. 2016) but overlapping (Graham 2015) constructs, may have been challenging although participants received definitions beforehand. These factors should be kept in mind when interpreting the results.

### Future directions

Future studies should also investigate the link between tiredness or fatigue and sexuality in individuals with various sexual orientations, and dyadic aspects should be explored by examining both partners. It would further be beneficial to investigate this link in individuals with chronic fatigue and in clinical samples, e.g., in individuals with chronic fatigue syndrome or with sexual desire or arousal disorders in daily life. In CFS patients, for example, the patient’s partner may place further stress on the topic of sexuality within the couple by perceiving the illness as avoidance (Blazquez et al. 2009). Previous studies hint at negative associations between symptoms of chronic fatigue and sexual experiences and behavior in CFS patients. For instance, one study reported an association between symptoms of fatigue and decreased sexual functioning and sexual desire in female Gulf War veterans (Gilhooly et al. 2001). Furthermore, the severity of fatigue was associated with decreased sexual satisfaction, sensuality and positive experience of sexual activities, as well as increased avoidance, in female patients suffering from chronic fatigue syndrome (CFS), which might be described as an extreme form of feeling constantly fatigued (Blazquez et al. 2008). Specifically, 85% of CFS patients (compared to 33% of healthy controls) wished to change an aspect of their sex life and 85% of CFS patients (compared to 7% of healthy controls) either felt exhausted following sexual activity or reported viewing sexual activity as a negative experience (Blazquez et al. 2008). In another study with women with CFS, of whom 58.7% were also diagnosed with fibromyalgia, 82.9% with Sjögren’s syndrome, and 65.5% with myofascial pain syndrome, a higher intensity of fatigue was also associated with a lower frequency of sexual intercourse, less communication about sexual problems, lower sexual sensuality, more avoidance of sexual contact, symptoms of vaginismus, and anorgasmia (Blazquez et al. 2015). In line with these findings, chronic illnesses that include fatigue as a symptom, such as fibromyalgia or rheumatoid arthritis, have been associated with decreased sexual desire and arousal (Alia et al. 2019; Bazzichi et al. 2012), and CFS has further been associated with vulvodynia symptoms (Arnold et al. 2007). In contrast, Vermeulen and Scholte (2004) found no significant differences in sexual functioning between female patients with CFS and female healthy controls, all in heterosexual relationships. However, in the control group (but not in the patient group), a higher fatigue score was significantly associated with a lower frequency of sexual fantasies, desire for sexual contact, sexual contact, and current satisfaction with one’s sex life. Further investigations into the link between CFS and sexual experiences and behavior using this study’s design may offer insights into intervention opportunities. Moreover, to gain further insights, future studies should investigate gender differences, as the present study shows differential effects between men and women regarding some of the links between tiredness, fatigue, sexual desire, and sexual arousal. Furthermore, considering the distinction between tiredness and fatigue applied by different samples may offer additional insights.

## Conclusion

The present study investigated bidirectional associations of tiredness, general fatigue, and physical fatigue with sexual desire and sexual arousal in daily life in heterosexual men and women. The results showed that higher sexual desire was associated with lower concurrent physical fatigue. Furthermore, higher tiredness was associated with higher subsequent sexual desire and arousal in men but not in women, who instead showed a slightly negative association. Moreover, although no significant main effect was found, higher sexual desire and arousal seem to predict lower subsequent physical fatigue in men and higher subsequent physical fatigue in women. Considering the Dual Control Model (Bancroft and Janssen 2000; Janssen and Bancroft 2007) tiredness and fatigue may be involved in eliciting and inhibiting sexual processes. Future research into these links in healthy as well as clinical samples may lead to new insights as well as treatment possibilities, ultimately improving quality of life.

## Supplementary Information

Below is the link to the electronic supplementary material.

Supplementary file1

## Declarations

### Transparency and openness statement

Research data and analysis code are available upon request. The research was not preregistered in an independent, institutional registry.
