# Supplementary material for: Tiredness/fatigue and sexuality in everyday life: Findings from an ecological momentary assessment
Source: J Neural Transm (Vienna). 2025 Sep 11;132(9):1417–30. doi: 10.1007/s00702-025-03008-9 (PMC12535510; doi:10.1007/s00702-025-03008-9)
Supplement: Supplementary file 1 — Supplementary file1 [file 702_2025_3008_MOESM1_ESM.docx]

Journal of Neural Transmission

**Online Resource 1**

Tiredness/fatigue and sexuality in everyday life: Findings from an ecological momentary assessment

Hanna M. Mües^1, 2^, Anja C. Feneberg^1, 3^, Charlotte Markert^4^, and Urs M. Nater^1^

1 University of Vienna, 2 Medical University of Vienna, 3 University of Münster, 4 Justus-Liebig-University Giessen

Correspondence concerning this article should be addressed to Urs M. Nater, Department of Clinical and Health Psychology, University of Vienna, Liebiggasse 5, 1010 Vienna, Austria. E-mail: [urs.nater@univie.ac.at](mailto:urs.nater@univie.ac.at). Telephone number: +43 1 4277 47220.

**Results**

Table S1 shows subjective fatigue levels reported at the baseline assessment. Table S2 shows reported mean levels of tiredness, general fatigue, and physical fatigue during the EMA phase. Details on descriptive statistics of sexual desire (*M* = 0.73, *SD* = 1.07, range: 0-4) and sexual arousal (*M* = 0.51, *SD* = 0.95, range: 0-4) during the EMA phase are provided in Mües et al. (in press).

**Table S1**

*Subjective fatigue levels using the Multidimensional Fatigue Inventory (MFI-20, 20 items; Smets et al., 1995) at the baseline assessment.*

|  | **Men (n = 31)** | | | **Women (n = 32)** | | | **Total (n = 63)** | | |
| --- | --- | --- | --- | --- | --- | --- | --- | --- | --- |
|  | ***M*** | ***SD*** | **Range** | ***M*** | ***SD*** | **Range** | ***M*** | ***SD*** | **Range** |
| **General fatigue** | 7.97 | 3.22 | 4.00-20.00 | 8.34 | 3.22 | 4.00-18.00 | 8.16 | 3.20 | 4.00-20.00 |
| **Physical fatigue** | 6.84 | 3.06 | 4.00-20.00 | 6.03 | 1.61 | 4.00-10.00 | 6.45 | 2.48 | 4.00-20.00 |
| **Reduced activity** | 8.03 | 3.10 | 4.00-20.00 | 7.27 | 3.18 | 4.00-16.00 | 7.64 | 3.14 | 4.00-20.00 |
| **Reduced motivation** | 6.97 | 3.26 | 4.00-20.00 | 6.31 | 2.28 | 4.00-14.00 | 6.63 | 2.79 | 4.00-20.00 |
| **Mental fatigue** | 8.43 | 3.37 | 4.00-20.00 | 8.16 | 4.02 | 4.0-18.0 | 8.30 | 3.68 | 4.0-20.0 |
| *Note*: Higher values indicate higher levels of the construct. | | | | | | | | | |

**Table S2**

*Tiredness, general fatigue, and physical fatigue during the EMA phase across individuals and measurements.*

|  | **Men (*n* = 2,192)** | **Women (*n* = 2,388)** | **Total (*n* = 4,580)** |
| --- | --- | --- | --- |
|  | ***M ± SD*** | ***M ± SD*** | ***M ± SD*** |
| **Tiredness** | 1.49 ± 1.20 | 1.64 ± 1.31 | 1.57 ± 1.26 |
| **General fatigue** | 1.43 ± 1.16 | 1.48 ± 1.27 | 1.45 ± 1.22 |
| **Physical fatigue** | 1.69 ± 1.08 | 1.91 ± 1.24 | 1.81 ± 1.17 |
| Note: Range: 0-4 (higher values indicate higher levels) for all variables. | | | |

Tables S3 to S32 show results of multilevel models.

**Table S3**

| *Three-level multilevel model for the effect of tiredness levels on sexual desire at the same measurement time point*. | | | |
| --- | --- | --- | --- |
|  | **Final Model** | | |
| *Predictors* | *Estimates* | *CI (95%)* | *p* |
| Intercept | 0.77 | 0.61 – 0.92 | **<0.001** |
| Tiredness | -0.03 | -0.07 – 0.01 | 0.109 |
| Age (in years) | -0.00 | -0.04 – 0.03 | 0.854 |
| PHQ-9 | 0.03 | -0.04 – 0.10 | 0.400 |
| Relationship duration (in months) | -0.00 | -0.01 – 0.00 | 0.635 |
| Relationship quality | -0.01 | -0.02 – 0.00 | 0.296 |
| Time since awakening (in hours) | 0.03 | 0.02 – 0.04 | **<0.001** |
| Previous event | 0.93 | 0.81 – 1.05 | **<0.001** |
| Autocorrelation | 0.14 | 0.10 – 0.17 | **<0.001** |
| Sleep quality | 0.00 | -0.00 – 0.00 | 0.468 |
| Gender | -0.31 | -0.52 – -0.09 | **0.005** |
| **Random Effects** | | | |
| σ^2^ | 0.81 |  |  |
| τ_00_ | 0.07 _Participant:Day_ |  |  |
|  | 0.13 _Participant_ |  |  |
| τ_11_ | 0.00 _Participant.Tiredness_ |  |  |
| ρ_01_ | 0.03 _Participant_ |  |  |
| ICC | 0.20 |  |  |
| N | 61 _Participant_ |  |  |
|  | 14 _Day_ |  |  |
| Observations | 3159 |  |  |
| Marginal R^2^ / Conditional R^2^ | 0.120 / 0.295 |  |  |

*Note.* CI: Confidence interval. PHQ-9: Depression Module from the Patient Health Questionnaire. Previous event: 0: no previous event, 1: at least one previous event occurred. Gender: 0: men, 1: women.**Table S4**

| *Three-level multilevel model for the effect of tiredness levels on sexual arousal at the same measurement time point*. | | | |
| --- | --- | --- | --- |
|  | **Final Model** | | |
| *Predictors* | *Estimates* | *CI (95%)* | *p* |
| Intercept | 0.53 | 0.40 – 0.66 | **<0.001** |
| Tiredness | -0.02 | -0.05 – 0.01 | 0.256 |
| Age (in years) | -0.02 | -0.06 – 0.01 | 0.112 |
| PHQ-9 | 0.03 | -0.03 – 0.08 | 0.363 |
| Relationship duration (in months) | 0.00 | -0.00 – 0.00 | 0.635 |
| Relationship quality | -0.00 | -0.01 – 0.01 | 0.737 |
| Time since awakening (in hours) | 0.02 | 0.01 – 0.02 | **<0.001** |
| Previous event | 1.13 | 1.03 – 1.24 | **<0.001** |
| Autocorrelation | 0.08 | 0.05 – 0.12 | **<0.001** |
| Sleep quality | 0.00 | -0.00 – 0.00 | 0.851 |
| Gender | -0.29 | -0.47 – -0.11 | **0.002** |
| **Random Effects** | | | |
| σ^2^ | 0.60 | | |
| τ_00_ | 0.03 _Participant:Day_ | | |
|  | 0.09 _Participant_ | | |
| τ_11_ | 0.00 _Participant.Tiredness_ | | |
| ρ_01_ | 0.43 _Participant_ | | |
| ICC | 0.18 | | |
| N | 61 _Participant_ | | |
|  | 14 _Day_ | | |
| Observations | 3159 | | |
| Marginal R^2^ / Conditional R^2^ | 0.160 / 0.311 | | |

*Note*. CI: Confidence interval. PHQ-9: depression subscale from the Patient Health Questionnaire. Previous event: 0: no previous event, 1: at least one previous event occurred. Gender: 0: men, 1: women.**Table S5**

| *Three-level multilevel model for the effect of general fatigue levels on sexual desire at the same measurement time point*. | | | |
| --- | --- | --- | --- |
|  | **Final Model** | | |
| *Predictors* | *Estimates* | *CI (95%)* | *p* |
| Intercept | 0.77 | 0.62 – 0.93 | **<0.001** |
| General fatigue | -0.01 | -0.05 – 0.02 | 0.414 |
| Age (in years) | -0.00 | -0.04 – 0.03 | 0.861 |
| PHQ-9 | 0.03 | -0.04 – 0.09 | 0.408 |
| Relationship duration (in months) | -0.00 | -0.01 – 0.00 | 0.637 |
| Relationship quality | -0.01 | -0.02 – 0.00 | 0.303 |
| Time since awakening (in hours) | 0.03 | 0.02 – 0.03 | **<0.001** |
| Previous event | 0.93 | 0.80 – 1.05 | **<0.001** |
| Autocorrelation | 0.14 | 0.10 – 0.17 | **<0.001** |
| Sleep quality | 0.00 | -0.00 – 0.00 | 0.420 |
| Gender | -0.31 | -0.52 – -0.09 | **0.005** |
| **Random Effects** | | | |
| σ^2^ | 0.81 |  |  |
| τ_00_ | 0.07 _Participant:Day_ |  |  |
|  | 0.13 _Participant_ |  |  |
| τ_11_ | 0.00 _Participant.General Fatigue_ |  |  |
| ρ_01_ | -0.22 _Participant_ |  |  |
| ICC | 0.19 |  |  |
| N | 61 _Participant_ |  |  |
|  | 14 _Day_ |  |  |
| Observations | 3159 |  |  |
| Marginal R^2^ / Conditional R^2^ | 0.120 / 0.291 |  |  |

*Note*. CI: Confidence interval. PHQ-9: Depression Module from the Patient Health Questionnaire. Previous event: 0: no previous event, 1: at least one previous event occurred. Gender: 0: men, 1: women.**Table S6**

| *Three-level multilevel model for the effect of general fatigue levels on sexual arousal at the same measurement time point*. | | | |
| --- | --- | --- | --- |
|  | **Final Model** | | |
| *Predictors* | *Estimates* | *CI (95%)* | *p* |
| Intercept | 0.54 | 0.41 – 0.67 | **<0.001** |
| General fatigue | -0.02 | -0.05 – 0.01 | 0.129 |
| Age (in years) | -0.02 | -0.06 – 0.01 | 0.111 |
| PHQ-9 | 0.03 | -0.03 – 0.09 | 0.298 |
| Relationship duration (in months) | 0.00 | -0.00 – 0.00 | 0.673 |
| Relationship quality | -0.00 | -0.01 – 0.01 | 0.692 |
| Time since awakening (in hours) | 0.02 | 0.01 – 0.02 | **<0.001** |
| Previous event | 1.13 | 1.02 – 1.24 | **<0.001** |
| Autocorrelation | 0.08 | 0.05 – 0.12 | **<0.001** |
| Sleep quality | 0.00 | -0.00 – 0.00 | 0.892 |
| Gender | -0.31 | -0.48 – -0.13 | **0.001** |
| **Random Effects** | | | |
| σ^2^ | 0.60 |  |  |
| τ_00_ | 0.03 _Participant:Day_ |  |  |
|  | 0.09 _Participant_ |  |  |
| τ_11_ | 0.00 _Participant.General Fatigue_ |  |  |
| ρ_01_ | 0.56 _Participant_ |  |  |
| ICC | 0.18 |  |  |
| N | 61 _Participant_ |  |  |
|  | 14 _Day_ |  |  |
| Observations | 3159 |  |  |
| Marginal R^2^ / Conditional R^2^ | 0.164 / 0.313 |  |  |

*Note*. CI: Confidence interval. PHQ-9: Depression Module from the Patient Health Questionnaire. Previous event: 0: no previous event, 1: at least one previous event occurred. Gender: 0: men, 1: women.**Table S7**

| *Three-level multilevel model for the effect of physical fatigue levels on sexual desire at the same measurement time point*. | | | |
| --- | --- | --- | --- |
|  | **Final Model** | | |
| *Predictors* | *Estimates* | *CI (95%)* | *p* |
| Intercept | 0.77 | 0.61 – 0.92 | **<0.001** |
| Physical fatigue | -0.03 | -0.07 – 0.00 | 0.079 |
| Age (in years) | -0.00 | -0.04 – 0.03 | 0.866 |
| PHQ-9 | 0.03 | -0.04 – 0.09 | 0.414 |
| Relationship duration (in months) | -0.00 | -0.01 – 0.00 | 0.619 |
| Relationship quality | -0.01 | -0.02 – 0.00 | 0.293 |
| Time since awakening (in hours) | 0.03 | 0.02 – 0.03 | **<0.001** |
| Previous event | 0.93 | 0.80 – 1.05 | **<0.001** |
| Autocorrelation | 0.14 | 0.10 – 0.17 | **<0.001** |
| Sleep quality | 0.00 | -0.00 – 0.00 | 0.472 |
| Gender | -0.31 | -0.52 – -0.09 | **0.005** |
| **Random Effects** | | | |
| σ^2^ | 0.81 |  |  |
| τ_00_ | 0.07 _Participant:Day_ |  |  |
|  | 0.13 _Participant_ |  |  |
| τ_11_ | 0.00 _Participant.Physical Fatigue_ |  |  |
| ρ_01_ | -0.03 _Participant_ |  |  |
| ICC | 0.20 |  |  |
| N | 61 _Participant_ |  |  |
|  | 14 _Day_ |  |  |
| Observations | 3159 |  |  |
| Marginal R^2^ / Conditional R^2^ | 0.119 / 0.295 |  |  |

*Note*. CI: Confidence interval. PHQ-9: Depression Module from the Patient Health Questionnaire. Previous event: 0: no previous event, 1: at least one previous event occurred. Gender: 0: men, 1: women.**Table S8**

| *Three-level multilevel model for the effect of physical fatigue levels on sexual arousal at the same measurement time point*. | | | |
| --- | --- | --- | --- |
|  | **Final Model** | | |
| *Predictors* | *Estimates* | *CI (95%)* | *p* |
| Intercept | 0.54 | 0.41 – 0.67 | **<0.001** |
| Physical fatigue | -0.02 | -0.05 – 0.02 | 0.328 |
| Age (in years) | -0.03 | -0.06 – 0.00 | 0.053 |
| PHQ-9 | 0.02 | -0.03 – 0.08 | 0.398 |
| Relationship duration (in months) | 0.00 | -0.00 – 0.01 | 0.469 |
| Relationship quality | -0.00 | -0.01 – 0.01 | 0.616 |
| Time since awakening (in hours) | 0.02 | 0.01 – 0.02 | **<0.001** |
| Previous event | 1.13 | 1.02 – 1.23 | **<0.001** |
| Autocorrelation | 0.08 | 0.05 – 0.12 | **<0.001** |
| Sleep quality | 0.00 | -0.00 – 0.00 | 0.844 |
| Gender | -0.31 | -0.48 – -0.13 | **0.001** |
| **Random Effects** | | | |
| σ^2^ | 0.60 |  |  |
| τ_00_ | 0.03 _Participant:Day_ |  |  |
|  | 0.09 _Participant_ |  |  |
| τ_11_ | 0.01 _Participant.Physical Fatigue_ |  |  |
| ρ_01_ | 0.38 _Participant_ |  |  |
| ICC | 0.18 |  |  |
| N | 61 _Participant_ |  |  |
|  | 14 _Day_ |  |  |
| Observations | 3159 |  |  |
| Marginal R^2^ / Conditional R^2^ | 0.165 / 0.318 |  |  |

*Note*. CI: Confidence interval. PHQ-9: Depression Module from the Patient Health Questionnaire. Previous event: 0: no previous event, 1: at least one previous event occurred. Gender: 0: men, 1: women.

**Table S9**

| *Two-level multilevel model for the effect of sexual desire on tiredness levels at the same measurement time point*. | | | |
| --- | --- | --- | --- |
|  | **Final Model** | | |
| *Predictors* | *Estimates* | *CI (95%)* | *p* |
| Intercept | 1.12 | 0.95 – 1.28 | **<0.001** |
| Sexual Desire | -0.03 | -0.07 – 0.01 | 0.117 |
| Age (in years) | 0.01 | -0.03 – 0.05 | 0.626 |
| PHQ-9 | 0.08 | 0.00 – 0.15 | **0.042** |
| Relationship duration (in months) | 0.00 | -0.00 – 0.01 | 0.136 |
| Relationship quality | -0.02 | -0.03 – -0.01 | **<0.001** |
| Time since awakening (in hours) | 0.13 | 0.13 – 0.14 | **<0.001** |
| Previous event | 0.06 | -0.07 – 0.20 | 0.344 |
| Autocorrelation | 0.33 | 0.30 – 0.37 | **<0.001** |
| Sleep quality | -0.00 | -0.00 – -0.00 | **0.042** |
| Gender | 0.20 | -0.04 – 0.43 | 0.100 |
| **Random Effects** | | | |
| σ^2^ | 0.90 |  |  |
| τ_00_ | 0.17 _Participant_ |  |  |
| τ_11_ | 0.00 _Participant.Sexual Desire_ |  |  |
| ρ_01_ | 0.05 _Participant_ |  |  |
| ICC | 0.16 |  |  |
| N | 61 _Participant_ |  |  |
| Observations | 3159 |  |  |
| Marginal R^2^ / Conditional R^2^ | 0.354 / 0.457 |  |  |

*Note*. CI: Confidence interval. PHQ-9: Depression Module from the Patient Health Questionnaire. Previous event: 0: no previous event, 1: at least one previous event occurred. Gender: 0: men, 1: women.

**Table S10**

| *Two-level multilevel model for the effect of sexual desire on general fatigue levels at the same measurement time point*. | | | |
| --- | --- | --- | --- |
|  | **Final Model** | | |
| *Predictors* | *Estimates* | *CI (95%)* | *p* |
| Intercept | 1.22 | 1.02 – 1.41 | **<0.001** |
| Sexual Desire | -0.02 | -0.05 – 0.02 | 0.415 |
| Age (in years) | 0.00 | -0.04 – 0.05 | 0.840 |
| PHQ-9 | 0.06 | -0.03 – 0.14 | 0.192 |
| Relationship duration (in months) | 0.00 | -0.00 – 0.01 | 0.151 |
| Relationship quality | -0.02 | -0.04 – -0.01 | **0.001** |
| Time since awakening (in hours) | 0.09 | 0.09 – 0.10 | **<0.001** |
| Previous event | -0.11 | -0.24 – 0.02 | 0.101 |
| Autocorrelation | 0.31 | 0.27 – 0.34 | **<0.001** |
| Sleep quality | -0.00 | -0.00 – 0.00 | 0.150 |
| Gender | 0.09 | -0.19 – 0.37 | 0.529 |
| **Random Effects** | | | |
| σ^2^ | 0.88 |  |  |
| τ_00_ | 0.24 _Participant_ |  |  |
| τ_11_ | 0.00 _Participant.Sexual Desire_ |  |  |
| ρ_01_ | 0.15 _Participant_ |  |  |
| ICC | 0.21 |  |  |
| N | 61 _Participant_ |  |  |
| Observations | 3159 |  |  |
| Marginal R^2^ / Conditional R^2^ | 0.259 / 0.418 |  |  |

*Note*. CI: Confidence interval. PHQ-9: Depression Module from the Patient Health Questionnaire. Previous event: 0: no previous event, 1: at least one previous event occurred. Gender: 0: men, 1: women.

**Table S11**

| *Two-level multilevel model for the effect of sexual desire on physical fatigue levels at the same measurement time point*. | | | |
| --- | --- | --- | --- |
|  | **Final Model** | | |
| *Predictors* | *Estimates* | *CI (95%)* | *p* |
| Intercept | 1.40 | 1.20 – 1.60 | **<0.001** |
| Sexual Desire | -0.04 | -0.08 – -0.00 | **0.048** |
| Age (in years) | -0.05 | -0.10 – 0.00 | 0.054 |
| PHQ-9 | 0.03 | -0.05 – 0.12 | 0.451 |
| Relationship duration (in months) | 0.00 | -0.00 – 0.01 | 0.455 |
| Relationship quality | -0.02 | -0.03 – -0.00 | **0.032** |
| Time since awakening (in hours) | 0.10 | 0.09 – 0.11 | **<0.001** |
| Previous event | 0.02 | -0.11 – 0.15 | 0.763 |
| Autocorrelation | 0.29 | 0.26 – 0.33 | **<0.001** |
| Sleep quality | -0.00 | -0.00 – 0.00 | 0.067 |
| Gender | 0.27 | -0.01 – 0.55 | 0.059 |
| **Random Effects** | | | |
| σ^2^ | 0.82 |  |  |
| τ_00_ | 0.25 _Participant_ |  |  |
| τ_11_ | 0.01 _Participant.Sexual Desire_ |  |  |
| ρ_01_ | 0.20 _Participant_ |  |  |
| ICC | 0.24 |  |  |
| N | 61 _Participant_ |  |  |
| Observations | 3159 |  |  |
| Marginal R^2^ / Conditional R^2^ | 0.243 / 0.426 |  |  |

*Note*. CI: Confidence interval. PHQ-9: Depression Module from the Patient Health Questionnaire. Previous event: 0: no previous event, 1: at least one previous event occurred. Gender: 0: men, 1: women.

**Table S12**

| *Two-level multilevel model for the effect of sexual arousal on tiredness levels at the same measurement time point*. | | | |
| --- | --- | --- | --- |
|  | **Final Model** | | |
| *Predictors* | *Estimates* | *CI (95%)* | *p* |
| Intercept | 1.12 | 0.95 – 1.28 | **<0.001** |
| Sexual Arousal | -0.03 | -0.08 – 0.01 | 0.152 |
| Age (in years) | 0.01 | -0.03 – 0.05 | 0.621 |
| PHQ-9 | 0.08 | 0.00 – 0.15 | **0.042** |
| Relationship duration (in months) | 0.00 | -0.00 – 0.01 | 0.137 |
| Relationship quality | -0.02 | -0.03 – -0.01 | **<0.001** |
| Time since awakening (in hours) | 0.13 | 0.13 – 0.14 | **<0.001** |
| Previous event | 0.07 | -0.07 – 0.21 | 0.313 |
| Autocorrelation | 0.33 | 0.30 – 0.37 | **<0.001** |
| Sleep quality | -0.00 | -0.00 – -0.00 | **0.041** |
| Gender | 0.20 | -0.04 – 0.43 | 0.102 |
| **Random Effects** | | | |
| σ^2^ | 0.90 |  |  |
| τ_00_ | 0.17 _Participant_ |  |  |
| τ_11_ | 0.00 _Participant.Sexual Arousal_ |  |  |
| ρ_01_ | 0.00 _Participant_ |  |  |
| ICC | 0.16 |  |  |
| N | 61 _Participant_ |  |  |
| Observations | 3159 |  |  |
| Marginal R^2^ / Conditional R^2^ | 0.354 / 0.457 |  |  |

*Note*. CI: Confidence interval. PHQ-9: Depression Module from the Patient Health Questionnaire. Previous event: 0: no previous event, 1: at least one previous event occurred. Gender: 0: men, 1: women.

**Table S13**

| *Two-level multilevel model for the effect of sexual arousal on general fatigue levels at the same measurement* time point. | | | |
| --- | --- | --- | --- |
|  | **Final Model** | | |
| *Predictors* | *Estimates* | *CI (95%)* | *p* |
| Intercept | 1.21 | 1.02 – 1.41 | **<0.001** |
| Sexual Arousal | -0.04 | -0.08 – 0.00 | 0.078 |
| Age (in years) | 0.00 | -0.04 – 0.05 | 0.853 |
| PHQ-9 | 0.05 | -0.03 – 0.14 | 0.218 |
| Relationship duration (in months) | 0.00 | -0.00 – 0.01 | 0.152 |
| Relationship quality | -0.02 | -0.04 – -0.01 | **0.001** |
| Time since awakening (in hours) | 0.09 | 0.09 – 0.10 | **<0.001** |
| Previous event | -0.08 | -0.21 – 0.05 | 0.234 |
| Autocorrelation | 0.31 | 0.27 – 0.34 | **<0.001** |
| Sleep quality | -0.00 | -0.00 – 0.00 | 0.153 |
| Gender | 0.09 | -0.19 – 0.36 | 0.537 |
| **Random Effects** | | | |
| σ^2^ | 0.88 |  |  |
| τ_00_ | 0.24 _Participant_ |  |  |
| ICC | 0.21 |  |  |
| N | 61 _Participant_ |  |  |
| Observations | 3159 |  |  |
| Marginal R^2^ / Conditional R^2^ | 0.259 / 0.417 |  |  |

*Note*. CI: Confidence interval. PHQ-9: Depression Module from the Patient Health Questionnaire. Previous event: 0: no previous event, 1: at least one previous event occurred. Gender: 0: men, 1: women.

**Table S14**

| *Two-level multilevel model for the effect of sexual arousal on physical fatigue levels at the same measurement time point.* | | | |
| --- | --- | --- | --- |
|  | **Final Model** | | |
| *Predictors* | *Estimates* | *CI (95%)* | *p* |
| Intercept | 1.42 | 1.22 – 1.62 | **<0.001** |
| Sexual Arousal | -0.03 | -0.08 – 0.01 | 0.159 |
| Age (in years) | -0.05 | -0.09 – 0.00 | 0.061 |
| PHQ-9 | 0.04 | -0.05 – 0.13 | 0.369 |
| Relationship duration (in months) | 0.00 | -0.00 – 0.01 | 0.512 |
| Relationship quality | -0.01 | -0.03 – -0.00 | **0.035** |
| Time since awakening (in hours) | 0.10 | 0.09 – 0.11 | **<0.001** |
| Previous event | 0.01 | -0.12 – 0.14 | 0.894 |
| Autocorrelation | 0.29 | 0.26 – 0.33 | **<0.001** |
| Sleep quality | -0.00 | -0.00 – -0.00 | **0.050** |
| Gender | 0.25 | -0.03 – 0.53 | 0.085 |
| **Random Effects** | | | |
| σ^2^ | 0.82 |  |  |
| τ_00_ | 0.25 _Participant_ |  |  |
| τ_11_ | 0.01 _Participant.Sexual Arousal_ |  |  |
| ρ_01_ | 0.19 _Participant_ |  |  |
| ICC | 0.24 |  |  |
| N | 61 _Participant_ |  |  |
| Observations | 3159 |  |  |
| Marginal R^2^ / Conditional R^2^ | 0.241 / 0.423 |  |  |

*Note*. CI: Confidence interval. PHQ-9: Depression Module from the Patient Health Questionnaire. Previous event: 0: no previous event, 1: at least one previous event occurred. Gender: 0: men, 1: women.

**Table S15**

| *Three-level multilevel model for the effect of tiredness levels at one measurement time point on sexual desire at the next time point*. | | | | |
| --- | --- | --- | --- | --- |
|  | **Final Model** | | | |
| *Predictors* | *Estimates* | *CI (95%)* | *p* | |
| Intercept | 0.78 | 0.62 – 0.93 | **<0.001** | |
| Tiredness | 0.08 | 0.01 – 0.14 | **0.023** | |
| Gender | -0.31 | -0.52 – -0.09 | **0.005** | |
| Age (in years) | -0.00 | -0.04 – 0.03 | 0.886 | |
| PHQ-9 | 0.03 | -0.04 – 0.09 | 0.447 | |
| Relationship duration (in months) | -0.00 | -0.01 – 0.00 | 0.680 | |
| Relationship quality | -0.01 | -0.02 – 0.01 | 0.327 | |
| Time since awakening (in hours) | 0.02 | 0.02 – 0.03 | **<0.001** | |
| Previous event | 0.92 | 0.80 – 1.05 | **<0.001** | |
| Autocorrelation | 0.14 | 0.10 – 0.17 | **<0.001** | |
| Sleep quality | 0.00 | -0.00 – 0.00 | 0.374 | |
| Interaction with Gender | -0.10 | -0.18 – -0.02 | **0.017** | |
| **Random Effects** | | | | |
| σ^2^ | 0.81 | | |  |
| τ_00_ | 0.06 _Participant:Day_ | | |  |
|  | 0.13 _Participant_ | | |  |
| τ_11_ | 0.01 _Participant.Lagged Tiredness_ | | |  |
| ρ_01_ | 0.11 _Participant_ | | |  |
| ICC | 0.20 | | |  |
| N | 61 _Participant_ | | |  |
|  | 14 _Day_ | | |  |
| Observations | 3159 | | |  |
| Marginal R^2^ / Conditional R^2^ | 0.120 / 0.295 | | |  |

*Note*. CI: Confidence interval. PHQ-9: Depression Module from the Patient Health Questionnaire. Previous event: 0: no previous event, 1: at least one previous event occurred. Gender: 0: men, 1: women.**Table S16**

| *Three-level multilevel model for the effect of tiredness levels at one measurement time point on sexual arousal at the next time point*. | | | |
| --- | --- | --- | --- |
|  | **Final Model** | | |
| *Predictors* | *Estimates* | *CI (95%)* | *p* |
| Intercept | 0.53 | 0.40 – 0.66 | **<0.001** |
| Tiredness | 0.05 | 0.00 – 0.10 | **0.032** |
| Gender | -0.28 | -0.46 – -0.10 | **0.003** |
| Age (in years) | -0.02 | -0.06 – 0.01 | 0.124 |
| PHQ-9 | 0.02 | -0.04 – 0.08 | 0.507 |
| Relationship duration (in months) | 0.00 | -0.00 – 0.00 | 0.642 |
| Relationship quality | -0.00 | -0.01 – 0.01 | 0.771 |
| Time since awakening (in hours) | 0.01 | 0.01 – 0.02 | **<0.001** |
| Previous event | 1.13 | 1.02 – 1.23 | **<0.001** |
| Autocorrelation | 0.08 | 0.05 – 0.12 | **<0.001** |
| Sleep quality | 0.00 | -0.00 – 0.00 | 0.741 |
| Interaction with Gender | -0.06 | -0.13 – -0.00 | **0.036** |
| **Random Effects** | | | |
| σ^2^ | 0.60 |  |  |
| τ_00_ | 0.03 _Participant:Day_ |  |  |
|  | 0.09 _Participant_ |  |  |
| ICC | 0.18 |  |  |
| N | 61 _Participant_ |  |  |
|  | 14 _Day_ |  |  |
| Observations | 3159 |  |  |
| Marginal R^2^ / Conditional R^2^ | 0.160 / 0.307 |  |  |

*Note*. CI: Confidence interval. PHQ-9: Depression Module from the Patient Health Questionnaire. Previous event: 0: no previous event, 1: at least one previous event occurred. Gender: 0: men, 1: women.**Table S17**

| *Three-level multilevel model for the effect of general fatigue levels at one measurement time point on sexual desire at the next time point*. | | | | |
| --- | --- | --- | --- | --- |
|  | **Final Model** | | | |
| *Predictors* | *Estimates* | *CI (95%)* | | *p* |
| Intercept | 0.78 | 0.63 – 0.94 | | **<0.001** |
| General fatigue | -0.00 | -0.04 – 0.04 | | 0.882 |
| Age (in years) | -0.00 | -0.04 – 0.03 | | 0.803 |
| PHQ-9 | 0.03 | -0.03 – 0.10 | | 0.313 |
| Relationship duration (in months) | -0.00 | -0.01 – 0.00 | | 0.578 |
| Relationship quality | -0.00 | -0.02 – 0.01 | | 0.350 |
| Time since awakening (in hours) | 0.02 | 0.02 – 0.03 | | **<0.001** |
| Previous event | 0.93 | 0.81 – 1.05 | | **<0.001** |
| Autocorrelation | 0.14 | 0.10 – 0.17 | | **<0.001** |
| Sleep quality | 0.00 | -0.00 – 0.00 | | 0.412 |
| Gender | -0.32 | -0.53 – -0.11 | | **0.003** |
| **Random Effects** | | | | |
| σ^2^ | 0.81 | |  |  |
| τ_00_ | 0.07 _Participant:Day_ | |  |  |
|  | 0.13 _Participant_ | |  |  |
| τ_11_ | 0.00 _Participant.Lagged General Fatigue_ | |  |  |
| ρ_01_ | -0.50 _Participant_ | |  |  |
| ICC | 0.20 | |  |  |
| N | 61 _Participant_ | |  |  |
|  | 14 _Day_ | |  |  |
| Observations | 3159 | |  |  |
| Marginal R^2^ / Conditional R^2^ | 0.121 / 0.294 | |  |  |

*Note*. CI: Confidence interval. PHQ-9: Depression Module from the Patient Health Questionnaire. Previous event: 0: no previous event, 1: at least one previous event occurred. Gender: 0: men, 1: women.

**Table S18**

| *Three-level multilevel model for the effect of general fatigue levels at one measurement time point on sexual arousal at the next time point*. | | | |
| --- | --- | --- | --- |
|  | **Final Model** | | |
| *Predictors* | *Estimates* | *CI (95%)* | *p* |
| Intercept | 0.53 | 0.40 – 0.66 | **<0.001** |
| General fatigue | 0.01 | -0.02 – 0.04 | 0.655 |
| Age (in years) | -0.02 | -0.06 – 0.01 | 0.125 |
| PHQ-9 | 0.02 | -0.04 – 0.08 | 0.507 |
| Relationship duration (in months) | 0.00 | -0.00 – 0.00 | 0.640 |
| Relationship quality | -0.00 | -0.01 – 0.01 | 0.766 |
| Time since awakening (in hours) | 0.01 | 0.01 – 0.02 | **<0.001** |
| Previous event | 1.13 | 1.03 – 1.24 | **<0.001** |
| Autocorrelation | 0.08 | 0.05 – 0.12 | **<0.001** |
| Sleep quality | 0.00 | -0.00 – 0.00 | 0.747 |
| Gender | -0.28 | -0.46 – -0.10 | **0.003** |
| **Random Effects** | | | |
| σ^2^ | 0.61 |  |  |
| τ_00_ | 0.03 _Participant:Day_ |  |  |
|  | 0.09 _Participant_ |  |  |
| ICC | 0.17 |  |  |
| N | 61 _Participant_ |  |  |
|  | 14 _Day_ |  |  |
| Observations | 3159 |  |  |
| Marginal R^2^ / Conditional R^2^ | 0.159 / 0.306 |  |  |

*Note*. CI: Confidence interval. PHQ-9: Depression Module from the Patient Health Questionnaire. Previous event: 0: no previous event, 1: at least one previous event occurred. Gender: 0: men, 1: women.

**Table S19**

| *Three-level multilevel model for the effect of physical fatigue levels at one measurement time point on sexual desire at the next time point*. | | | | |
| --- | --- | --- | --- | --- |
|  | **Final Model** | | | |
| *Predictors* | *Estimates* | *CI (95%)* | | *p* |
| Intercept | 0.79 | 0.64 – 0.94 | | **<0.001** |
| Physical fatigue | 0.03 | -0.01 – 0.07 | | 0.179 |
| Age (in years) | -0.01 | -0.04 – 0.03 | | 0.783 |
| PHQ-9 | 0.04 | -0.03 – 0.10 | | 0.287 |
| Relationship duration (in months) | -0.00 | -0.01 – 0.00 | | 0.551 |
| Relationship quality | -0.01 | -0.02 – 0.00 | | 0.305 |
| Time since awakening (in hours) | 0.02 | 0.02 – 0.03 | | **<0.001** |
| Previous event | 0.93 | 0.80 – 1.05 | | **<0.001** |
| Autocorrelation | 0.14 | 0.10 – 0.17 | | **<0.001** |
| Sleep quality | 0.00 | -0.00 – 0.00 | | 0.280 |
| Gender | -0.33 | -0.54 – -0.12 | | **0.002** |
| **Random Effects** | | | | |
| σ^2^ | 0.81 | |  |  |
| τ_00_ | 0.06 _Participant:Day_ | |  |  |
|  | 0.13 _Participant_ | |  |  |
| τ_11_ | 0.01 _Participant.Lagged Physical Fatigue_ | |  |  |
| ρ_01_ | -0.36 _Participant_ | |  |  |
| ICC | 0.20 | |  |  |
| N | 61 _Participant_ | |  |  |
|  | 14 _Day_ | |  |  |
| Observations | 3159 | |  |  |
| Marginal R^2^ / Conditional R^2^ | 0.122 / 0.298 | |  |  |

*Note*. CI: Confidence interval. PHQ-9: Depression Module from the Patient Health Questionnaire. Previous event: 0: no previous event, 1: at least one previous event occurred. Gender: 0: men, 1: women.**Table S20**

| *Three-level multilevel model for the effect of physical fatigue levels at one measurement time point on sexual arousal at the next time point*. | | | | |
| --- | --- | --- | --- | --- |
|  | **Final Model** | | | |
| *Predictors* | *Estimates* | *CI (95%)* | | *p* |
| Intercept | 0.52 | 0.39 – 0.65 | | **<0.001** |
| Physical fatigue | 0.03 | -0.00 – 0.07 | | 0.082 |
| Age (in years) | -0.02 | -0.06 – 0.01 | | 0.121 |
| PHQ-9 | 0.02 | -0.04 – 0.07 | | 0.577 |
| Relationship duration (in months) | 0.00 | -0.00 – 0.00 | | 0.648 |
| Relationship quality | -0.00 | -0.01 – 0.01 | | 0.633 |
| Time since awakening (in hours) | 0.01 | 0.01 – 0.02 | | **<0.001** |
| Previous event | 1.13 | 1.02 – 1.23 | | **<0.001** |
| Autocorrelation | 0.08 | 0.05 – 0.12 | | **<0.001** |
| Sleep quality | 0.00 | -0.00 – 0.00 | | 0.639 |
| Gender | -0.26 | -0.44 – -0.08 | | **0.005** |
| **Random Effects** | | | | |
| σ^2^ | 0.60 | |  |  |
| τ_00_ | 0.03 _Participant:Day_ | |  |  |
|  | 0.10 _Participant_ | |  |  |
| τ_11_ | 0.01 _Participant.Lagged Physical Fatigue_ | |  |  |
| ρ_01_ | 0.27 _Participant_ | |  |  |
| ICC | 0.18 | |  |  |
| N | 61 _Participant_ | |  |  |
|  | 14 _Day_ | |  |  |
| Observations | 3159 | |  |  |
| Marginal R^2^ / Conditional R^2^ | 0.157 / 0.311 | |  |  |

*Note*. CI: Confidence interval. PHQ-9: Depression Module from the Patient Health Questionnaire. Previous event: 0: no previous event, 1: at least one previous event occurred. Gender: 0: men, 1: women.**Table S21**

| *Two-level multilevel model for the effect of sexual desire at one measurement time point on tiredness levels at the next time point*. | | | |
| --- | --- | --- | --- |
|  | **Final Model** | | |
| *Predictors* | *Estimates* | *CI (95%)* | *p* |
| Intercept | 1.12 | 0.95 – 1.28 | **<0.001** |
| Sexual Desire | 0.03 | -0.01 – 0.07 | 0.174 |
| Age (in years) | 0.01 | -0.03 – 0.05 | 0.604 |
| PHQ-9 | 0.08 | 0.01 – 0.15 | **0.035** |
| Relationship duration (in months) | 0.00 | -0.00 – 0.01 | 0.109 |
| Relationship quality | -0.02 | -0.03 – -0.01 | **<0.001** |
| Time since awakening (in hours) | 0.13 | 0.13 – 0.14 | **<0.001** |
| Previous event | 0.01 | -0.11 – 0.14 | 0.831 |
| Autocorrelation | 0.33 | 0.30 – 0.37 | **<0.001** |
| Sleep quality | -0.00 | -0.00 – -0.00 | **0.027** |
| Gender | 0.21 | -0.02 – 0.44 | 0.079 |
| **Random Effects** | | | |
| σ^2^ | 0.90 | | |
| τ_00_ | 0.17 _Participant_ | | |
| τ_11_ | 0.01 _Participant.Lagged Sexual Desire_ | | |
| ρ_01_ | -0.22 _Participant_ | | |
| ICC | 0.16 | | |
| N | 61 _Participant_ | | |
| Observations | 3159 | | |
| Marginal R^2^ / Conditional R^2^ | 0.355 / 0.459 | | |

*Note*. CI: Confidence interval. PHQ-9: Depression Module from the Patient Health Questionnaire. Previous event: 0: no previous event, 1: at least one previous event occurred. Gender: 0: men, 1: women.

**Table S22**

| *Two-level multilevel model for the effect of sexual desire at one measurement time point on general fatigue levels at the next time point*. | | | |
| --- | --- | --- | --- |
|  | **Final Model** | | |
| *Predictors* | *Estimates* | *CI (95%)* | *p* |
| Intercept | 1.22 | 1.03 – 1.42 | **<0.001** |
| Sexual Desire | 0.00 | -0.05 – 0.05 | 0.997 |
| Age (in years) | 0.01 | -0.04 – 0.05 | 0.821 |
| PHQ-9 | 0.06 | -0.03 – 0.14 | 0.206 |
| Relationship duration (in months) | 0.00 | -0.00 – 0.01 | 0.149 |
| Relationship quality | -0.02 | -0.04 – -0.01 | **0.001** |
| Time since awakening (in hours) | 0.09 | 0.08 – 0.10 | **<0.001** |
| Previous event | -0.14 | -0.26 – -0.01 | **0.032** |
| Autocorrelation | 0.31 | 0.27 – 0.34 | **<0.001** |
| Sleep quality | -0.00 | -0.00 – 0.00 | 0.164 |
| Gender | 0.08 | -0.19 – 0.36 | 0.560 |
| **Random Effects** | | | |
| σ^2^ | 0.87 |  |  |
| τ_00_ | 0.24 _Participant_ |  |  |
| τ_11_ | 0.01 _Participant.Lagged Sexual_ Desire |  |  |
| ρ_01_ | -0.03 _Participant_ |  |  |
| ICC | 0.22 |  |  |
| N | 61 _Participant_ |  |  |
| Observations | 3159 |  |  |
| Marginal R^2^ / Conditional R^2^ | 0.258 / 0.422 |  |  |

*Note*. CI: Confidence interval. PHQ-9: Depression Module from the Patient Health Questionnaire. Previous event: 0: no previous event, 1: at least one previous event occurred. Gender: 0: men, 1: women.**Table S23**

| *Two-level multilevel model for the effect of sexual desire at one measurement time point on physical fatigue levels at the next time point*. | | | |
| --- | --- | --- | --- |
|  | **Final Model** | | |
| *Predictors* | *Estimates* | *CI (95%)* | *p* |
| Intercept | 1.42 | 1.22 – 1.62 | **<0.001** |
| Sexual Desire | -0.03 | -0.07 – 0.02 | 0.302 |
| Gender | 0.26 | -0.03 – 0.54 | 0.077 |
| Age (in years) | -0.04 | -0.09 – 0.01 | 0.085 |
| PHQ-9 | 0.04 | -0.05 – 0.12 | 0.414 |
| Relationship duration (in months) | 0.00 | -0.00 – 0.01 | 0.599 |
| Relationship quality | -0.01 | -0.03 – -0.00 | **0.047** |
| Time since awakening (in hours) | 0.10 | 0.09 – 0.11 | **<0.001** |
| Previous event | -0.03 | -0.15 – 0.09 | 0.626 |
| Autocorrelation | 0.29 | 0.26 – 0.33 | **<0.001** |
| Sleep quality | -0.00 | -0.00 – -0.00 | **0.043** |
| Interaction with Gender | 0.09 | 0.02 – 0.16 | **0.008** |
| **Random Effects** | | | |
| σ^2^ | 0.82 | | |
| τ_00_ | 0.25 _Participant_ | | |
| ICC | 0.23 | | |
| N | 61 _Participant_ | | |
| Observations | 3159 | | |
| Marginal R^2^ / Conditional R^2^ | 0.240 / 0.418 | | |

*Note*. CI: Confidence interval. PHQ-9: Depression Module from the Patient Health Questionnaire. Previous event: 0: no previous event, 1: at least one previous event occurred. Gender: 0: men, 1: women.

**Table S24**

| *Two-level multilevel model for the effect of sexual arousal at one measurement time point on tiredness levels at the next time point*. | | | |
| --- | --- | --- | --- |
|  | **Final Model** | | |
| *Predictors* | *Estimates* | *CI (95%)* | *p* |
| Intercept | 1.11 | 0.94 – 1.27 | **<0.001** |
| Sexual Arousal | 0.00 | -0.04 – 0.05 | 0.835 |
| Age (in years) | 0.01 | -0.03 – 0.05 | 0.516 |
| PHQ-9 | 0.08 | 0.01 – 0.16 | **0.024** |
| Relationship duration (in months) | 0.00 | -0.00 – 0.01 | 0.141 |
| Relationship quality | -0.02 | -0.03 – -0.01 | **<0.001** |
| Time since awakening (in hours) | 0.13 | 0.13 – 0.14 | **<0.001** |
| Previous event | 0.03 | -0.10 – 0.16 | 0.631 |
| Autocorrelation | 0.33 | 0.30 – 0.37 | **<0.001** |
| Sleep quality | -0.00 | -0.00 – -0.00 | **0.042** |
| Gender | 0.22 | -0.01 – 0.45 | 0.062 |
| **Random Effects** | | | |
| σ^2^ | 0.90 | | |
| τ_00_ | 0.17 _Participant_ | | |
| τ_11_ | 0.01 _Participant.Lagged Sexual Arousal_ | | |
| ρ_01_ | -0.48 _Participant_ | | |
| ICC | 0.16 | | |
| N | 61 _Participant_ | | |
| Observations | 3159 | | |
| Marginal R^2^ / Conditional R^2^ | 0.356 / 0.459 | | |

*Note*. CI: Confidence interval. PHQ-9: Depression Module from the Patient Health Questionnaire. Previous event: 0: no previous event, 1: at least one previous event occurred. Gender: 0: men, 1: women.**Table S25**

| *Two-level multilevel model for the effect of sexual arousal at one measurement time point on general fatigue levels at the next time point*. | | | |
| --- | --- | --- | --- |
|  | **Final Model** | | |
| *Predictors* | *Estimates* | *CI (95%)* | *p* |
| Intercept | 1.22 | 1.03 – 1.42 | **<0.001** |
| Sexual Arousal | -0.01 | -0.07 – 0.04 | 0.587 |
| Age (in years) | 0.01 | -0.04 – 0.05 | 0.810 |
| PHQ-9 | 0.06 | -0.03 – 0.14 | 0.206 |
| Relationship duration (in months) | 0.00 | -0.00 – 0.01 | 0.173 |
| Relationship quality | -0.02 | -0.04 – -0.01 | **0.001** |
| Time since awakening (in hours) | 0.09 | 0.08 – 0.10 | **<0.001** |
| Previous event | -0.12 | -0.25 – 0.00 | 0.054 |
| Autocorrelation | 0.31 | 0.27 – 0.34 | **<0.001** |
| Sleep quality | -0.00 | -0.00 – 0.00 | 0.168 |
| Gender | 0.08 | -0.19 – 0.36 | 0.561 |
| **Random Effects** | | | |
| σ^2^ | 0.87 | | |
| τ_00_ | 0.24 _Participant_ | | |
| τ_11_ | 0.01 _Participant.Lagged Sexual Arousal_ | | |
| ρ_01_ | -0.13 _Participant_ | | |
| ICC | 0.22 | | |
| N | 61 _Participant_ | | |
| Observations | 3159 | | |
| Marginal R^2^ / Conditional R^2^ | 0.257 / 0.422 | | |

*Note*. CI: Confidence interval. PHQ-9: Depression Module from the Patient Health Questionnaire. Previous event: 0: no previous event, 1: at least one previous event occurred. Gender: 0: men, 1: women.

**Table S26**

| *Two-level multilevel model for the effect of sexual arousal at one measurement time point on physical fatigue levels at the next time point*. | | | |
| --- | --- | --- | --- |
|  | **Final Model** | | |
| *Predictors* | *Estimates* | *CI (95%)* | *p* |
| Intercept | 1.42 | 1.22 – 1.62 | **<0.001** |
| Sexual Arousal | -0.04 | -0.09 – 0.01 | 0.162 |
| Gender | 0.26 | -0.03 – 0.54 | 0.077 |
| Age (in years) | -0.04 | -0.09 – 0.01 | 0.085 |
| PHQ-9 | 0.04 | -0.05 – 0.12 | 0.415 |
| Relationship duration (in months) | 0.00 | -0.00 – 0.01 | 0.604 |
| Relationship quality | -0.01 | -0.03 – -0.00 | **0.047** |
| Time since awakening (in hours) | 0.10 | 0.09 – 0.11 | **<0.001** |
| Previous event | -0.02 | -0.14 – 0.10 | 0.699 |
| Autocorrelation | 0.29 | 0.26 – 0.33 | **<0.001** |
| Sleep quality | -0.00 | -0.00 – -0.00 | **0.048** |
| Interaction with Gender | 0.10 | 0.02 – 0.18 | **0.015** |
| **Random Effects** | | | |
| σ^2^ | 0.82 | | |
| τ_00_ | 0.25 _Participant_ | | |
| ICC | 0.23 | | |
| N | 61 _Participant_ | | |
| Observations | 3159 | | |
| Marginal R^2^ / Conditional R^2^ | 0.240 / 0.418 | | |

*Note*. CI: Confidence interval. PHQ-9: Depression Module from the Patient Health Questionnaire. Previous event: 0: no previous event, 1: at least one previous event occurred. Gender: 0: men, 1: women.

**Table S27**

| *Two-level multilevel models for the effect of occurrence of previous sexual activity on subsequent tiredness*. | | | |
| --- | --- | --- | --- |
|  | **Final Model** | | |
| *Predictors* | *Estimates* | *CI (95%)* | *p* |
| Intercept | 1.11 | 0.94 – 1.28 | **<0.001** |
| Previous sexual activity | 0.03 | -0.10 – 0.17 | 0.621 |
| Age (in years) | 0.01 | -0.03 – 0.05 | 0.641 |
| PHQ_9 | 0.07 | 0.00 – 0.14 | **0.049** |
| Relationship duration (in months) | 0.00 | -0.00 – 0.01 | 0.139 |
| Relationship quality | -0.02 | -0.04 – -0.01 | **<0.001** |
| Time since awakening | 0.13 | 0.13 – 0.14 | **<0.001** |
| Autocorrelation | 0.33 | 0.30 – 0.37 | **<0.001** |
| Sleep quality | -0.00 | -0.00 – -0.00 | **0.038** |
| Gender | 0.21 | -0.02 – 0.44 | 0.074 |
| **Random Effects** | | | |
| σ^2^ | 0.90 | | |
| τ_00_ _Participant_ | 0.17 | | |
| τ_11_ _Participant.Previous sexual activity_ | 0.02 | | |
| ρ_01_ _Participant_ | -0.63 | | |
| ICC | 0.16 | | |
| N _Participant_ | 61 | | |
| Observations | 3159 | | |
| Marginal R^2^ / Conditional R^2^ | 0.357 / 0.458 | | |

*Note*. CI: Confidence interval. PHQ-9: Depression Module from the Patient Health Questionnaire. Previous event: 0: no previous event, 1: at least one previous event occurred. Gender: 0: men, 1: women.

**Table S28**

| *Two-level multilevel models for the effect of tiredness on subsequent occurrence of sexual activity*. | | | |
| --- | --- | --- | --- |
|  | **Final Model** | | |
| *Predictors* | *Odds Ratios* | *CI (95%)* | *p* |
| Intercept | 0.10 | 0.09 – 0.13 | **<0.001** |
| Tiredness | 0.90 | 0.81 – 1.01 | 0.085 |
| Age (in years) | 1.01 | 0.96 – 1.06 | 0.688 |
| PHQ_9 | 1.15 | 1.06 – 1.25 | **0.001** |
| Relationship duration (in months) | 1.00 | 0.99 – 1.01 | 0.967 |
| Relationship quality | 1.01 | 1.00 – 1.03 | 0.064 |
| Time since awakening | 1.02 | 0.99 – 1.04 | 0.203 |
| Sleep quality | 1.00 | 0.99 – 1.01 | 0.654 |
| Gender | 0.51 | 0.39 – 0.68 | **<0.001** |
| **Random Effects** | | | |
| σ^2^ | 3.29 | | |
| τ_00_ _Participant_ | 0.05 | | |
| ICC | 0.01 | | |
| N _Participant_ | 61 | | |
| Observations | 4272 | | |
| Marginal R^2^ / Conditional R^2^ | 0.037 / 0.051 | | |

*Note*. CI: Confidence interval. PHQ-9: Depression Module from the Patient Health Questionnaire. Previous event: 0: no previous event, 1: at least one previous event occurred. Gender: 0: men, 1: women.

**Table S29**

| *Two-level multilevel models for the effect of occurrence of previous sexual activity on subsequent general fatigue*. | | | |
| --- | --- | --- | --- |
|  | **Final Model** | | |
| *Predictors* | *Estimates* | *CI (95%)* | *p* |
| Intercept | 1.22 | 1.03 – 1.42 | **<0.001** |
| Previous sexual activity | -0.12 | -0.25 – 0.00 | 0.053 |
| Age (in years) | 0.00 | -0.04 – 0.05 | 0.875 |
| PHQ_9 | 0.05 | -0.03 – 0.14 | 0.213 |
| Relationship duration (in months) | 0.00 | -0.00 – 0.01 | 0.138 |
| Relationship quality | -0.02 | -0.04 – -0.01 | **0.001** |
| Time since awakening | 0.09 | 0.08 – 0.10 | **<0.001** |
| Autocorrelation | 0.31 | 0.27 – 0.34 | **<0.001** |
| Sleep quality | -0.00 | -0.00 – 0.00 | 0.150 |
| Gender | 0.08 | -0.19 – 0.36 | 0.564 |
| **Random Effects** | | | |
| σ^2^ | 0.88 | | |
| τ_00_ _Participant_ | 0.24 | | |
| τ_11_ _Participant.Previous sexual activity_ | 0.00 | | |
| ρ_01_ _Participant_ | -0.99 | | |
| ICC | 0.21 | | |
| N _Participant_ | 61 | | |
| Observations | 3159 | | |
| Marginal R^2^ / Conditional R^2^ | 0.260 / 0.417 | | |

*Note*. CI: Confidence interval. PHQ-9: Depression Module from the Patient Health Questionnaire. Previous event: 0: no previous event, 1: at least one previous event occurred. Gender: 0: men, 1: women.

**Table S30**

| *Two-level multilevel models for the effect of general fatigue on subsequent occurrence of sexual activity*. | | | |
| --- | --- | --- | --- |
|  | **Final Model** | | |
| *Predictors* | *Odds Ratios* | *CI (95%)* | *p* |
| Intercept | 0.11 | 0.09 – 0.13 | **<0.001** |
| General fatigue | 0.97 | 0.86 – 1.10 | 0.643 |
| Age (in years) | 1.01 | 0.96 – 1.06 | 0.682 |
| PHQ_9 | 1.15 | 1.06 – 1.25 | **0.001** |
| Relationship duration (in months) | 1.00 | 0.99 – 1.01 | 0.968 |
| Relationship quality | 1.01 | 1.00 – 1.03 | 0.063 |
| Time since awakening | 1.01 | 0.99 – 1.03 | 0.459 |
| Sleep quality | 1.00 | 1.00 – 1.01 | 0.545 |
| Gender | 0.51 | 0.39 – 0.68 | **<0.001** |
| **Random Effects** | | | |
| σ^2^ | 3.29 | | |
| τ_00_ _Participant_ | 0.05 | | |
| ICC | 0.01 | | |
| N _Participant_ | 61 | | |
| Observations | 4272 | | |
| Marginal R^2^ / Conditional R^2^ | 0.034 / 0.048 | | |

*Note*. CI: Confidence interval. PHQ-9: Depression Module from the Patient Health Questionnaire. Previous event: 0: no previous event, 1: at least one previous event occurred. Gender: 0: men, 1: women.

**Table S31**

| *Two-level multilevel models for the effect of occurrence of previous sexual activity on subsequent physical fatigue*. | | | |
| --- | --- | --- | --- |
|  | **Final Model** | | |
| *Predictors* | *Estimates* | *CI (95%)* | *p* |
| Intercept | 1.42 | 1.22 – 1.62 | **<0.001** |
| Previous sexual activity | -0.03 | -0.16 – 0.11 | 0.695 |
| Age (in years) | -0.04 | -0.09 – 0.01 | 0.083 |
| PHQ_9 | 0.04 | -0.05 – 0.12 | 0.424 |
| Relationship duration (in months) | 0.00 | -0.00 – 0.01 | 0.619 |
| Relationship quality | -0.01 | -0.03 – -0.00 | **0.048** |
| Time since awakening | 0.10 | 0.09 – 0.11 | **<0.001** |
| Autocorrelation | 0.29 | 0.26 – 0.33 | **<0.001** |
| Sleep quality | -0.00 | -0.00 – -0.00 | **0.048** |
| Gender | 0.25 | -0.03 – 0.54 | 0.078 |
| **Random Effects** | | | |
| σ^2^ | 0.82 | | |
| τ_00_ _Participant_ | 0.25 | | |
| τ_11_ _Participant.Previous sexual activity_ | 0.04 | | |
| ρ_01_ _Participant_ | 0.04 | | |
| ICC | 0.24 | | |
| N _Participant_ | 61 | | |
| Observations | 3159 | | |
| Marginal R^2^ / Conditional R^2^ | 0.238 / 0.419 | | |

*Note*. CI: Confidence interval. PHQ-9: Depression Module from the Patient Health Questionnaire. Previous event: 0: no previous event, 1: at least one previous event occurred. Gender: 0: men, 1: women.**Table S32**

| *Two-level multilevel models for the effect of physical fatigue on subsequent occurrence of sexual activity*. | | | |
| --- | --- | --- | --- |
|  | **Final Model** | | |
| *Predictors* | *Odds Ratios* | *CI (95%)* | *p* |
| Intercept | 0.10 | 0.09 – 0.13 | **<0.001** |
| Physical fatigue | 0.83 | 0.70 – 0.98 | **0.030** |
| Gender | 0.52 | 0.39 – 0.69 | **<0.001** |
| Age (in years) | 1.01 | 0.96 – 1.06 | 0.689 |
| PHQ_9 | 1.15 | 1.06 – 1.25 | **0.001** |
| Relationship duration (in months) | 1.00 | 0.99 – 1.01 | 0.968 |
| Relationship quality | 1.01 | 1.00 – 1.03 | 0.064 |
| Time since awakening | 1.01 | 0.99 – 1.04 | 0.317 |
| Sleep quality | 1.00 | 1.00 – 1.01 | 0.538 |
| Interaction with gender | 1.26 | 1.00 – 1.58 | **0.048** |
| **Random Effects** | | | |
| σ^2^ | 3.29 | | |
| τ_00_ _Participant_ | 0.05 | | |
| ICC | 0.02 | | |
| N _Participant_ | 61 | | |
| Observations | 4272 | | |
| Marginal R^2^ / Conditional R^2^ | 0.037 / 0.052 | | |

*Note*. CI: Confidence interval. PHQ-9: Depression Module from the Patient Health Questionnaire. Previous event: 0: no previous event, 1: at least one previous event occurred. Gender: 0: men, 1: women.

References

Mües, H. M., Markert, C., Feneberg, A. C., & Nater, U. M. (in press). Too stressed for sex? Associations between stress and sex in daily life. *Psychoneuroendocrinology*. <https://doi.org/10.1016/j.psyneuen.2025.107583>

Smets, E. M. A., Garssen, B., Bonke, B., & De Haes, J. C. J. M. (1995). The Multidimensional Fatigue Inventory (MFI) psychometric qualities of an instrument to assess fatigue. *Journal of Psychosomatic Research*, *39*(3), 315-325. <https://doi.org/10.1016/0022-3999(94)00125-o>
